# The Late Endosomal HOPS Complex Anchors Active G-Protein Signaling Essential for Pathogenesis in *Magnaporthe oryzae*

**DOI:** 10.1371/journal.ppat.1003527

**Published:** 2013-08-01

**Authors:** Ravikrishna Ramanujam, Meredith E. Calvert, Poonguzhali Selvaraj, Naweed I. Naqvi

**Affiliations:** 1 Temasek Life Sciences Laboratory, National University of Singapore, Singapore, Singapore; 2 School of Biological Sciences, Nanyang Technological University, Singapore, Singapore; 3 Department of Biological Sciences, National University of Singapore, Singapore, Singapore.; Purdue University, United States of America

## Abstract

In *Magnaporthe oryzae*, the causal ascomycete of the devastating rice blast disease, the conidial germ tube tip must sense and respond to a wide array of requisite cues from the host in order to switch from polarized to isotropic growth, ultimately forming the dome-shaped infection cell known as the appressorium. Although the role for G-protein mediated Cyclic AMP signaling in appressorium formation was first identified almost two decades ago, little is known about the spatio-temporal dynamics of the cascade and how the signal is transmitted through the intracellular network during cell growth and morphogenesis. In this study, we demonstrate that the late endosomal compartments, comprising of a PI3P-rich (Phosphatidylinositol 3-phosphate) highly dynamic tubulo-vesicular network, scaffold active MagA/Gα_S_, Rgs1 (a GAP for MagA), Adenylate cyclase and Pth11 (a non-canonical GPCR) in the likely absence of AKAP-like anchors during early pathogenic development in *M. oryzae*. Loss of HOPS component Vps39 and consequently the late endosomal function caused a disruption of adenylate cyclase localization, cAMP signaling and appressorium formation. Remarkably, exogenous cAMP rescued the appressorium formation defects associated with *VPS39* deletion in *M. oryzae*. We propose that sequestration of key G-protein signaling components on dynamic late endosomes and/or endolysosomes, provides an effective molecular means to compartmentalize and control the spatio-temporal activation and rapid downregulation (likely via vacuolar degradation) of cAMP signaling amidst changing cellular geometry during pathogenic development in *M. oryzae*.

## Author Summary

*Magnaporthe oryzae*, a filamentous fungus, causes the devastating and economically important blast disease in rice and related species. It produces asexual spores, conidia, which can switch to a disease-causing mode of development in response to host and environmental cues. Activation of a highly conserved cell-signaling cascade, the G-proteins/Cyclic AMP pathway, has been shown to be important for pathogenic development in *M. oryzae*. In this study, we demonstrate that the late endosomal compartment is a highly dynamic tubulo-vesicular network that apart from membrane trafficking also anchors active G-protein signaling components in *M. oryzae* and therefore regulates the biogenesis and spatio-temporal dynamics of cAMP signaling. Furthermore, utilizing specific mutant strains compromised for late endosomal function we specifically demonstrate the importance of signal anchoring by late endosomes during the establishment and spread of blast disease in rice and barley. Thus, we propose that anchoring of G-protein signaling on late endosomes endows *M. oryzae* with the ability to specifically activate, integrate and achieve modularity of signaling and eventually control the robustness as well as the temporal duration of signaling responses critical for pathogenesis.

## Introduction

Eukaryotes, ranging from yeasts to multicellular metazoans, interact with their environment, constantly sampling it for physico-chemical signals or cues for proper growth and development. Extracellular ligands or stimuli detected by membrane bound GPCR (G- protein coupled receptors) are transmitted to the cell interior by heterotrimeric (αβγ) guanine nucleotide binding proteins (G-proteins), which function as intrinsic molecular switches. Ligand activated receptors promote the exchange of GDP to GTP on cognate Gα_S_ subunit, triggering its dissociation from the βγ, thereby rendering it active to signal downstream [1]. Both Gα_S_·GTP and Gβγ moieties subsequently propagate the signal through a host of downstream effectors, which include ion channels, adenylate cyclases, phosphodiesterases and phospholipases [2], [3]. The foremost of these is adenylate cyclase that synthesizes the second messenger Cyclic AMP (cAMP) from ATP. Active signaling by the Gα_S_·GTP persists until the bound GTP is hydrolyzed to GDP, by the intrinsic GTPase activity of Gα_S_, permitting Gα_S_ to re-associate with Gβγ to form an inactive complex, and thereby commencing a fresh cycle of signaling [1], [4].

The duration of active signaling by Gα_S_ is dependent on the nucleotide state of the Gα_S_ subunit. In addition to the intrinsic GTPase activity of Gα_S_, the hydrolysis of GTP to GDP is regulated and fine-tuned by RGS proteins, which ultimately influence the duration of active G-protein signaling [5], [6]. RGS proteins are highly conserved negative regulators of G-protein signaling and function by stabilizing the switch regions on the Gα_S_ subunit which undergo a conformational change upon GTP hydrolysis, thereby accelerating the rate of hydrolysis of bound GTP to GDP on the active Gα_S_ subunit [7], [8], [9], [10], [11].

In conjunction with upstream GPCRs, heterotrimeric G-proteins regulate a wide-range of cellular and developmental pathways including aspects of nutrient sensing, mating and pathogenesis in fungi and yeasts [12]. For example, they are involved in sensing glucose in *S. cerevisiae* and *S. pombe*; amino acids in *C. albicans* and *C. neoformans* [13], [14], [15], [16] and carbon sources in *N. crassa*, *A. nidulans* and *B. cinerea* [17], [18], [19]. Additionally, G-proteins are involved in the pheromone response pathway in *S. cerevisiae* [20] and regulate mating and fruiting body formation in ascomycete fungi such as *A. nidulans, N. crassa and M. oryzae*, as well as basidiomycetes such as *U. maydis* and *C. neoformans* [12], [21], [22], [23], [24], [25], [26], [27], [28], [29].

G-protein signaling also plays an important role in regulating disease causing ability and progression (infection structure formation, pathogenicity factors or toxin production) in many plant and animal pathogens. A wide range of filamentous phyto-pathogens such as *C. parasitica*, *M. oryzae*, *U. maydis*, *B. cinerea*, *F. oxysporum*, *C. trifolii*, *S. nodorum* and *A. alternata* disrupted for trimeric G-proteins are defective in pathogenicity [12], [23], [24], [25], [30], [31], [32], [33], [34]. Similarly, functional G-protein cascades play a crucial role in establishing pathogenicity in human pathogenic fungi like *C. neoformans A. fumigatus* and *C. albicans* [14], [16], [27], [28], [35], [36].

In general, when and where G-protein signaling modules are activated in the context of cell shape and cellular geometry has a profound implication on downstream responses and behavior. As a result, cells have evolved complex molecular mechanisms to compartmentalize and thereby control the spatial and temporal dynamics of signaling pathways. In mammalian cells, signal compartmentalization is achieved by sequestration and/or anchoring of key regulators and effectors on different subcellular organelles, supramolecular complexes or scaffold proteins such as AKAPs (A-kinase anchoring proteins) [37], [38]. Anchoring of signaling proteins endows the cell with a host of advantages, which include the ability to specifically target and activate signaling pathways or components within a defined region of the cell, thereby preventing non-specific or off-target effects. Furthermore, signal anchoring *per se* can help the cell integrate and achieve modularity of signaling responses and ultimately regulate the amplitude and temporal duration of signaling in a precise manner [39].

The endosomal system represents a highly dynamic and biochemically specialized continuum of membranous tubulo-vesicular compartments involved primarily in the sorting, delivery and degradation of a diverse array of cargoes (macromolecules, biological signals) and maintaining cellular homeostasis. Cargo can enter the endolysosomal pathway either through endocytosis or internalization that takes place at the cell surface or through transport from internal biosynthetic pathways. The endosomal system is broadly categorized into early, late/MVBs (multi vesicular bodies) and recycling compartments. Each compartment is inherently programmed to carry out specific cellular functions. Early endosomes represent the first sites within which internalized components are deposited. Upon internalization, the components may be recycled back to the plasma membrane or may enter a late endosome/MVB for transport into the vacuole/lysosome. Early endosomal compartments usually mature over time, become increasingly acidic, and undergo homotypic fusions to form either late endosomes or accumulate intraluminal vesicles to develop MVBs. As they mature, the early endosomes first lose the Rab5 GTPase and eventually acquire Rab7 GTPase thereby becoming competent to undergo fusion with the degradative vacuole [40], [41]. Endosomes are known to serve functions beyond cargo sorting. Recent findings suggest that endosomal compartments additionally function as signaling anchors/platforms, integrating membrane trafficking and intracellular signal transduction [42], [43], [44], [45], [46], [47].

Upon hydration, the asexual spores produced by *M. oryzae* germinate to form slender germ tubes, which respond to inductive surface cues such as hydrophobicity and hardness, to undergo morphogenic transitions to ultimately form an infection cell known as the appressorium. One of the most critical steps during early pathogenic differentiation in *M. oryzae* is the “hooking” stage wherein the germ tube tip switches from polarized to isotropic growth (3–4 hpi) and subsequently forms an appressorium (8–12 hpi). It is thought that hooking constitutes a “recognition phase” in which surface characteristics are sensed and assessed prior to commitment to appressorium formation [48], [49].

A host of genetic studies carried out over the last decade have established that components of the G-protein/cAMP signaling cascade are highly conserved and play an important role in regulating various aspects of asexual development, appressorium formation, disease establishment and progression in *M. oryzae* [23], [50], [51], [52], [53], [54], [55]. However, little is known about the subcellular dynamics and/or the spatio-temporal organization of the critical cAMP-signaling module. In this study, we determine the intracellular localization of the G-protein cascade in *M. oryzae* with particular emphasis on the dynamics of active signaling components during the crucial stages of host surface sensing and recognition. We further demonstrate that the late endosomes represent important sites of active G-protein signaling and that the disruption of late endosomal integrity results in the disruption of host surface based signaling and subsequent disease causing ability. Our results unravel an elegant adaptive mechanism in *M. oryzae* for anchoring and trafficking of G-protein signaling components for cAMP synthesis during initiation of infection-related growth and development.

## Results

### Inductive surface induced dynamics of Rgs1

RGS proteins (Regulator of G-protein Signaling) play a crucial role in controlling the intensity and duration of early G-protein signaling [1], [6]. We utilized live cell imaging to gain insight into the spatial and temporal dynamics of Rgs1 in *M. oryzae*, particularly during the early events of surface recognition and appressorium morphogenesis.

**Figure 1:**
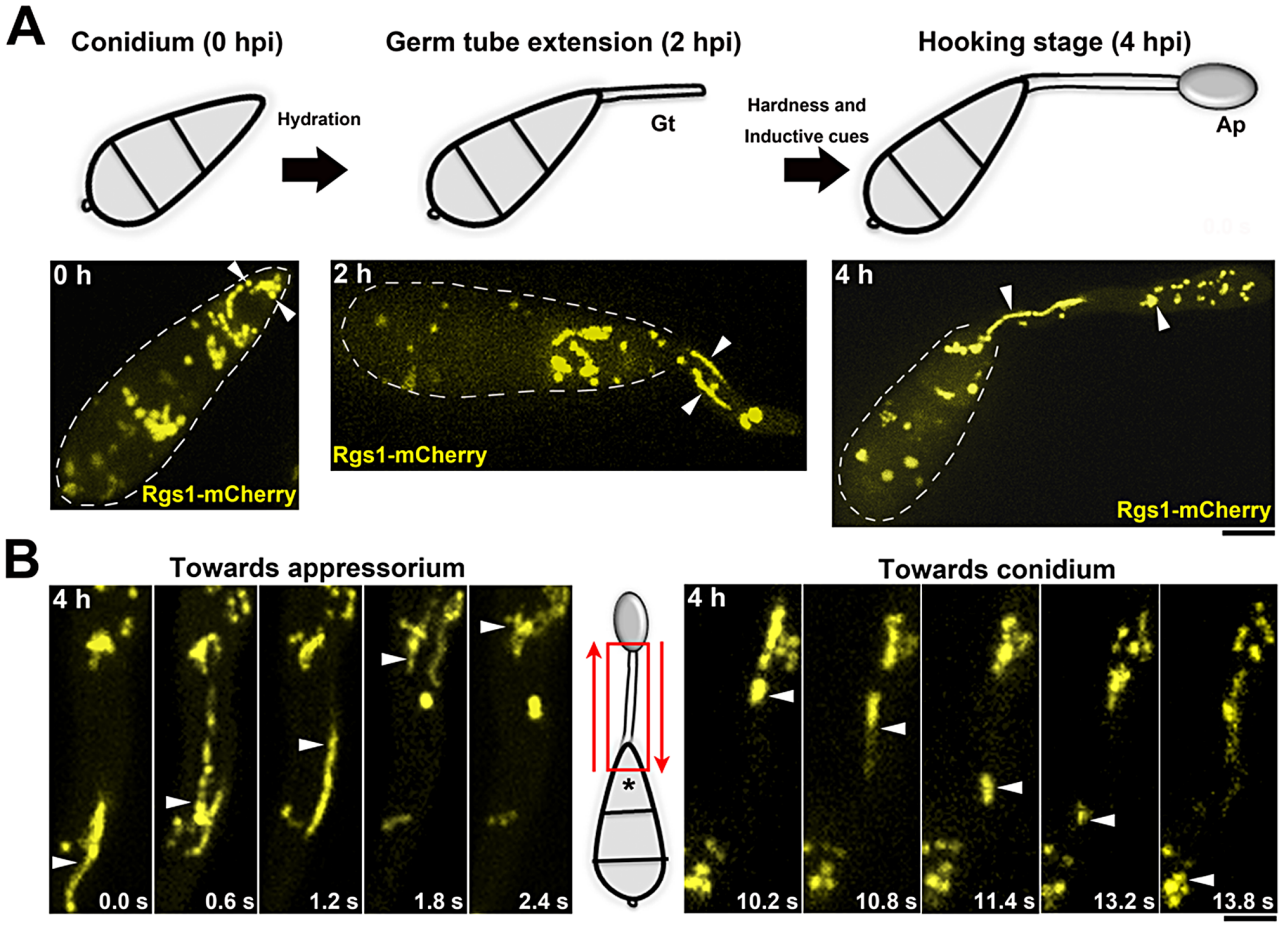
Subcellular localization and spatio-temporal dynamics of the negative regulator of G-protein signaling (Rgs1) in *M.* *oryzae*. (**A**) A schematic of the time line and key morphogenic transitions exhibited by *M. oryzae* in response to inductive cues. Gt: developing germ tube; and Ap: incipient appressorium at the hooking stage. Lower panels are maximum intensity Z-projections of five confocal slices, 0.5 µm each, of Rgs1-mCherry (pseudocolored yellow) strain at the corresponding time points. Arrowheads indicate punctate (0 h) and tubulo-vesicular compartments of Rgs1 in the developing germ tube (2 h) and the incipient appressorium (4 h). Scale bar, 10 µm. (**B**) Bi-directional mobility of Rgs1-mCherry tubulo-vesicular compartments, 4 hpi. Red arrows indicate the direction of movement of Rgs1 compartments towards either the appressorium or the terminal cell of the conidium (asterisk). Elapsed time indicated in seconds. Images are in maximum intensity Z-projections of five confocal stacks, 0.5 µm each. See also Video S1. Scale bar = 10 µm.

Imaging was performed on germinating conidia at 2 and 4 hpi on an inductive surface. Ungerminated conidia (0 hpi) were used as a control. Rgs1-mCherry (mC) was present on less dynamic punctate vesicles in ungerminated conidia; however it localized to highly dynamic and distinct tubulo-vesicular structures during the stages of germination and hooking (Figure 1A).

We next asked if the formation of the tubulo-vesicular structures or the observed dynamics of the Rgs1 compartments was influenced by inductive surface characteristics. On a non-inductive surface (1% agar) that does not support appressorium formation [54], Rgs1-mC failed to localize to dynamic tubulo-vesicular structures, but was predominantly vacuolar or present as small puncta in developing germ tubes (Figure S1A in Text S1). Likewise, the *pth11*Δ mutant, which is defective in sensing and responding to inductive surface cues [55], lacked such tubulo-vesicular structures and showed a predominantly vacuolar Rgs1-mC signal in the conidial cells (Figure S1B in Text S1). Rgs1-mC localized to tubulo-vesicular compartments at identical time points in the WT control strain.

Moreover, the dynamic mobility of Rgs1-containing structures was not random but inherently bidirectional, with a subset of the population moving towards the incipient appressorium, while a few punctae trafficked towards the terminal cell of the conidium (Figure 1B; Video S1).

Taken together, we conclude that in response to inductive surface cues, such as hardness and hydrophobicity, Rgs1-mC localizes to dynamic tubulo-vesicular compartments that traverse bi-directionally during appressorium initiation in *M. oryzae*. In the absence of such inductive cues or signals, as in the *pth11*Δ strain, Rgs1 is predominantly targeted to the vacuoles.

### Rgs1 compartments represent sites of active G-protein signaling

**Figure 2:**
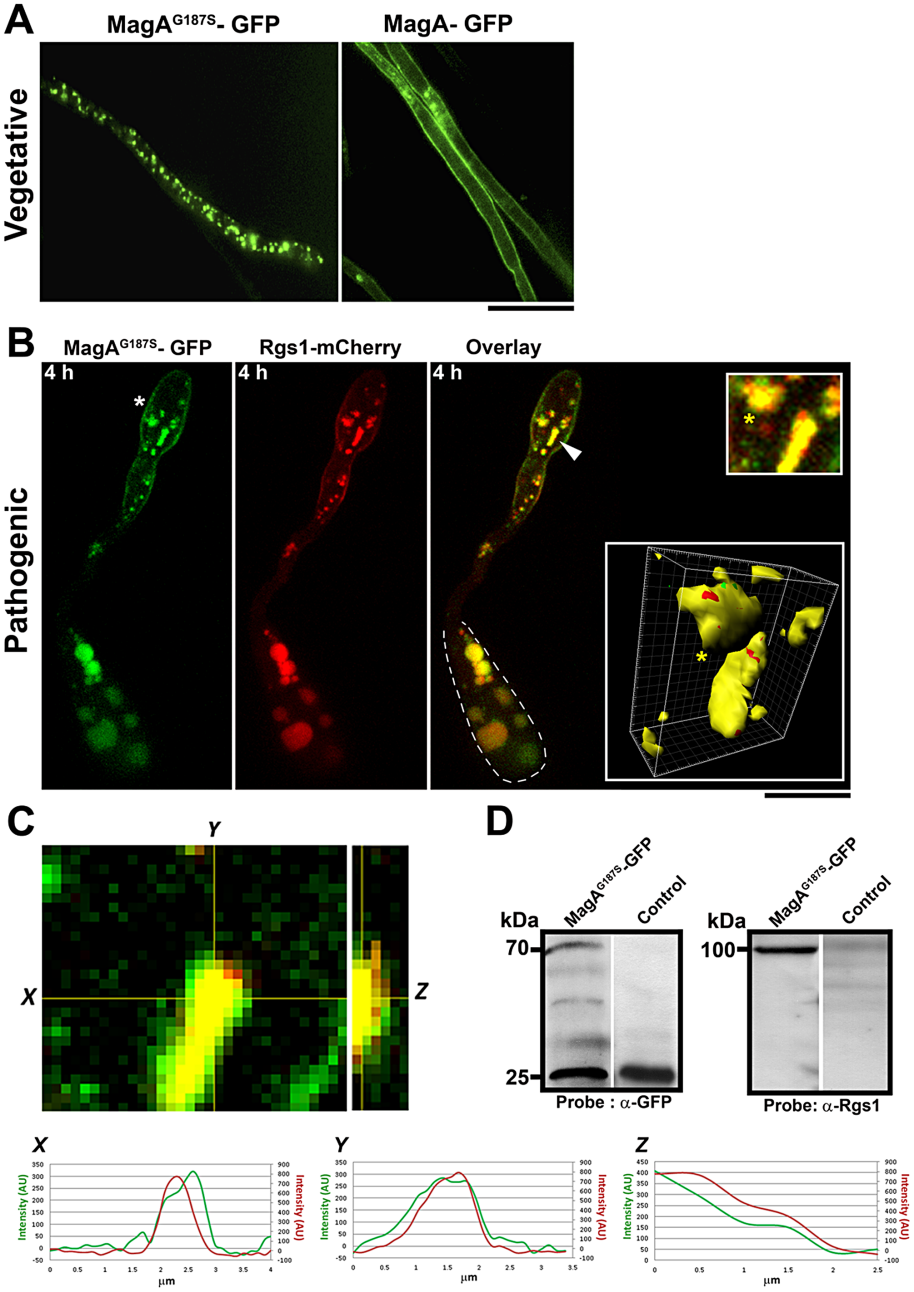
Rgs1 co-localizes with active Gα_S_/MagA during appressorium initiation or early pathogenic development. (**A**) Subcellular localization of MagA^G187S^-GFP and MagA-GFP during vegetative growth. Mycelia from the indicated strains were subjected to confocal microscopy. Single plane images are depicted. Scale bar, 10 µm. (**B**) Colocalization of constitutive active MagA^G187S^-GFP (green) and Rgs1-mCherry (red) compartments during the hooking stage. MagA^G187S^-GFP localizes to cytoplasmic vesicular structures and the plasma membrane (asterisk). The arrow highlights compartments co-populated by MagA^G187S^-GFP and Rgs1-mCherry and the yellow asterisk highlights the same compartment in the inset and the corresponding 3D surface rendering (panels on the far right). The dotted line demarcates the respective conidium. Images in panel B are maximum intensity Z-projections of six confocal stacks, 0.5 µm each. Scale bar equals 10 µm. (**C**) Single plane images of merged channels and corresponding orthogonal view of entire stack showing the co-localization of MagA^G187S^-GFP and Rgs1-mCherry. Representative intensity profiles were obtained for each channel from a single slice along the indicated axes (X and Y) and from the corresponding transverse section of the stack (Z) using Fiji software (**D**) MagA^G187S^ physically interacts with Rgs1. Western blot analysis from a GFP-trap based pull down experiment showing the specific interaction between MagA^G187S^ and Rgs1 during pathogenic differentiation. After probing for MagA^G187S^ -GFP with α-GFP antibody, the same membrane was stripped and re-probed using α-Rgs1 antibody. Total protein from a cytosolic-GFP strain served as a negative control. Equal concentrations of protein from both the strains were used for the pull down experiment. Scale bar = 10 µm.

We have previously demonstrated that Rgs1 physically interacts with and negatively regulates the GTP-bound MagA *in vitro* [54]. In order to understand the physiological relevance of Rgs1 localization to the dynamic compartments especially in the context of active G-protein signaling, a *M. oryzae* strain expressing both Rgs1-mC and a GFP-tagged constitutively active MagA/Gα_S_ subunit [54] (*MagA^G187S^*) was generated by insertion of the GFP coding sequence in the alpha B-alpha C loop of MagA [56]. The wild-type MagA-GFP was used as control. Remarkably, we observed a differential pattern of localization between the constitutively active MagA^G187S^ variant and the wild-type MagA in the vegetative mycelium. The active MagA^G187S^-GFP predominantly localized to distinct vesicles, whereas the wild-type MagA-GFP was primarily plasma membrane bound (Figure 2A).

Next, we tested if the active MagA^G187S^ co-localizes with Rgs1 during early pathogenesis and specifically during the hooking stage (4 hpi). Indeed, active MagA^G187S^-GFP vesicles co-localized with Rgs1-mC (Figure 2B), as determined by line scan analysis (Figure 2C) and verified quantitatively by Pearson's coefficient analysis (value = 0.56) (Figure S2A in Text S1). Furthermore, utilizing a GFP-trap based pull-down assay with whole cell extracts from MagA^G187S^-GFP and a control strain (cytosolic-GFP expressing strain), we found that MagA^G187S^ physically interacts with Rgs1 (Figure 2D). We conclude that Rgs1-containing vesicles and tubules represent sites of active G-protein signaling in *M. oryzae*. Furthermore, we infer that a preferential association exists between Rgs1 and the GTP-bound MagA on such dynamic structures during early stages of surface recognition and appressorium initiation.

### Rgs1 physically interacts with Pth11, a non-canonical GPCR

**Figure 3:**
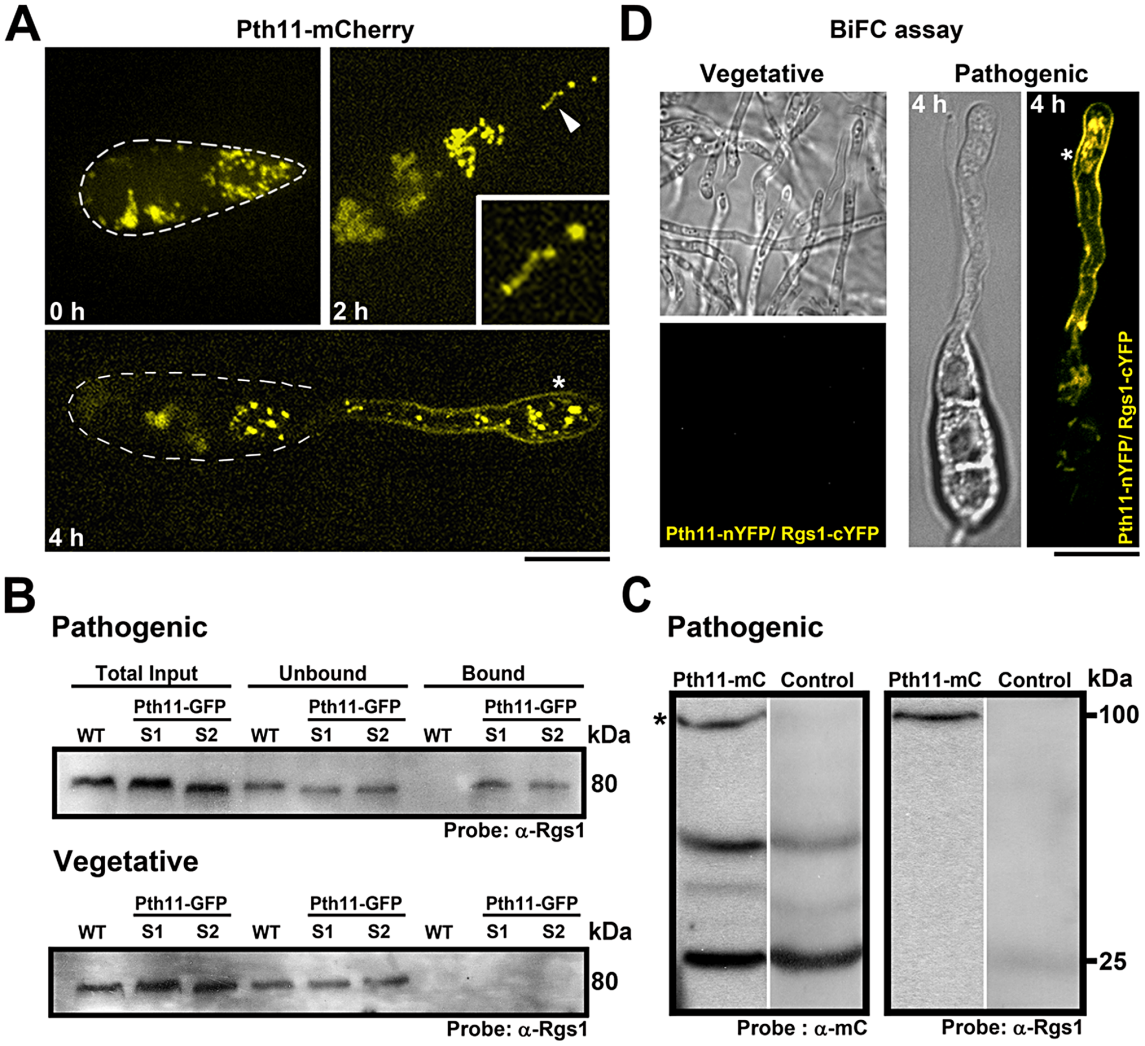
Localization of Pth11 and its interaction with Rgs1 in *M.* *oryzae*. (**A**) Conidia expressing Pth11-mCherry (pseudocolored yellow) were inoculated on an inductive surface and imaged at the indicated time-points. The inset in the 2 h panel highlights a tubulo-vesicular structure containing Pth11-mCherry. The asterisk depicts the association of Pth11 with the plasma membrane. The dotted line delineates the conidial boundary. Images are maximum intensity Z-projections of five confocal stacks, measuring 0.5 µm each. Scale bar, 10 µm. (**B**) Pth11 physically interacts with Rgs1. Immunoblots from a pull down experiment depicting the specific interaction between Pth11 and Rgs1 during appressorium initiation. Pth11 physically interacted with Rgs1 during pathogenic differentiation and failed to interact with Rgs1 during vegetative growth. In each case, equal concentrations of protein from both the strains were used for the pull down experiment. Total protein from an untagged wild-type strain served as a negative control in B. S1 and S2 represent two independent Pth11-GFP expressing strains. (**C**) An RFP-trap experiment depicting the specific interaction between Pth11-mCherry and Rgs1. After probing for Pth11-mCherry with α-mCherry antibody, the same membrane was stripped and re-probed using α-Rgs1 antibody. Total protein from a cytosolic-mCherry expressing strain served as a negative control in C. (**D**) Confocal microscopy based imaging of a BiFC experiment illustrating the *in-vivo* interaction between Pth11-nYFP and Rgs1-cYFP in the vegetative mycelium and at 4 h post inoculation on an inductive surface (right; DIC in left panel and YFP in right panel). Asterisk indicates YFP signal and thus likely interaction at the plasma membrane. Images are single plane images captured by a confocal microscope. Scale bar, 10 µm.

Pth11, the CFEM-domain containing non-canonical receptor, is predicted to function upstream of the cAMP signaling pathway in *M. oryzae* [55]. Given that active MagA and Rgs1 colocalized on dynamic structures during early stages of appressorium initiation, we sought to investigate the subcellular localization of Pth11 at this crucial stage of pathogenic development. We found that in a manner identical to Rgs1, a significant fraction of Pth11-mCherry localized to intracellular punctate and/or dynamic tubular structures. In addition, Pth11-mC also localized to the plasma membrane of the germ tube and incipient appressorium (Figure 3A).

Although predicted to be a GPCR involved in cAMP pathway [55], there is no evidence supporting the interaction of Pth11 with requisite downstream G-protein signaling components. To assess such an interaction, we performed GFP-trap based pull-down assays using whole cell lysates from a Pth11-GFP strain. Relevant immuno-precipitated or wild type control samples were analyzed by western blotting using α-Rgs1 antibody. Remarkably, Pth11 physically interacted with Rgs1 during the hooking stage (4 hpi) and not during vegetative growth (Figure 3B). We further confirmed the physical interaction between Pth11 and Rgs1 by performing an RFP-trap based pull-down with total cell extracts from the Pth11-mCherry and a control strain (cytosolic mCherry expressing strain) (Figure 3C). Additionally, in a BiFC (Bimolecular Fluorescence Complementation) assay Pth11-nYFP and Rgs1-cYFP interacted *in vivo* during early pathogenesis (2 and 4 hpi) as judged by the YFP signal detected in the plasma membrane and punctate structures within the germ tube (Figure 3D; Figure S3A in Text S1). Such an interaction between Pth11-nYFP and Rgs1-cYFP was not apparent in the control strains (Figure S3B) or during vegetative growth (Figure 3D). Taken together, we conclude that Pth11, active MagA and Rgs1 localize to dynamic tubulo-vesicular compartments. In addition, Pth11 physically interacts with Rgs1 specifically during appressorium initiation or germ tube hooking.

### Rgs1 localization to PI3P-containing compartments is dependent on sustained PI3 kinase activity

To determine if Rgs1-mC vesicles formed a part of the endolysosomal network, we performed a colocalization analysis with the fluorescent probe lysotracker green (LTG) that marks acidic endosomal compartments. A large number of structures with diverse morphologies were stained by LTG, whereas only a small population colocalized with Rgs1-mC, indicating that Rgs1 compartments formed a sub-population of the endosomal network (Figure S4A in Text S1).

**Figure 4:**
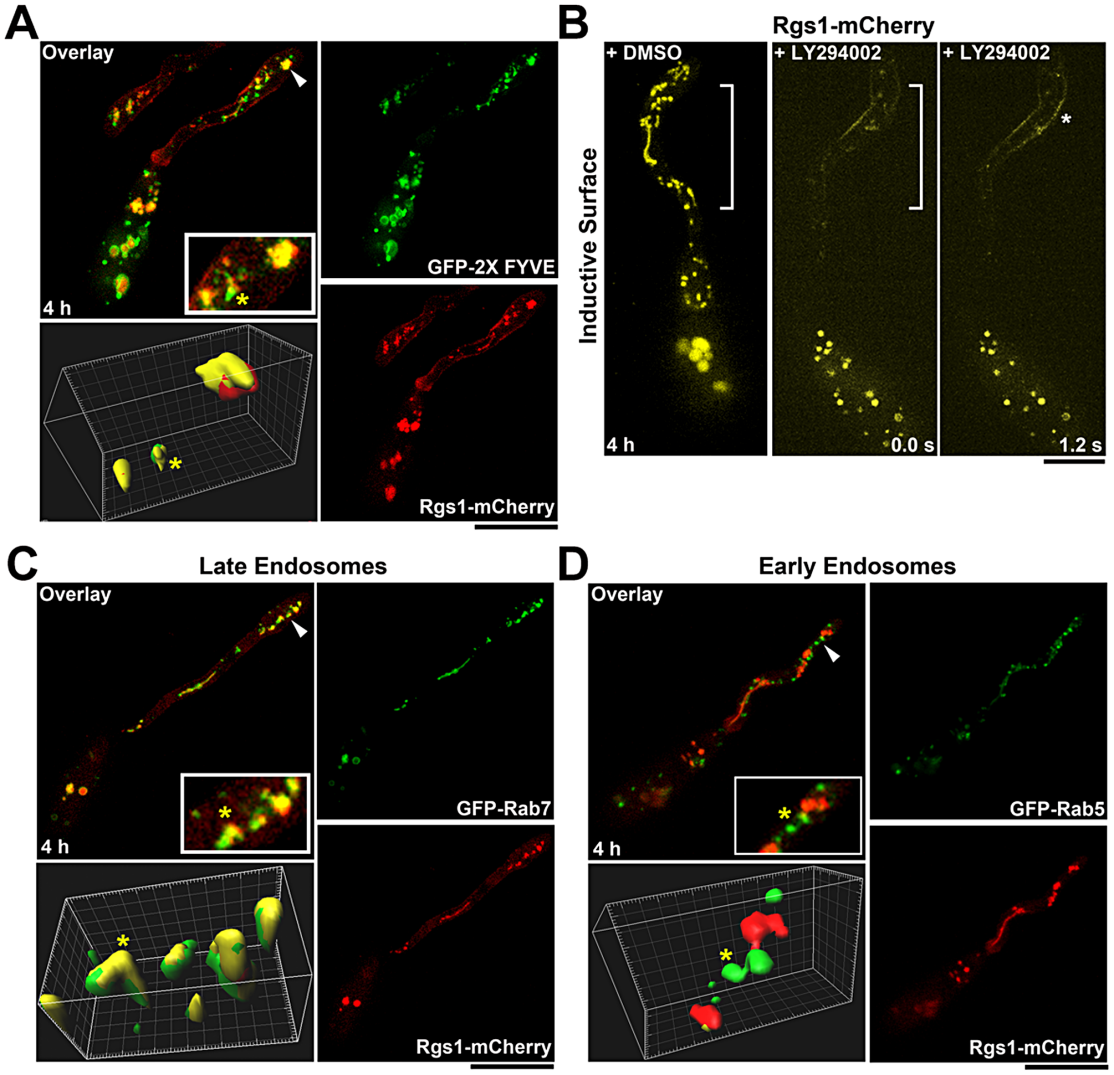
Identity and integrity of the tubulo-vesicular compartments that harbor Rgs1. (**A**) Rgs1-mCherry colocalizes with certain PI3P-rich endosomal compartments (marked by GFP-FYVE probe). Arrowheads mark the regions magnified in the inset and surface rendered in 3D. (**B**) Inhibition of PI3 kinase activity by LY294002 results in the loss of Rgs1-mCherry (pseudocoloured yellow) association with tubulo-vesicular structures. Asterisk indicates Rgs1 accumulation along the plasma membrane of the incipient appressorium. DMSO treated sample was used a control. The regions of the germ tube lacking the Rgs1-mC compartments structures are bracketed. LY294002 treatment was carried out 4 hpi. The elapsed time is indicated in seconds (**C**) GFP-Rab7 colocalizes with Rgs1-mCherry during early stages of pathogenesis. The arrowhead depicts the region magnified in the inset and surface rendered in 3D. (**D**) The early endosomal marker Rab5 does not colocalize with Rgs1-mCherry. Arrowheads mark the regions magnified in the inset and surface rendered in 3D. Images are maximum intensity Z-projections of five confocal stacks (0.5 µm each) and the yellow asterisk, in the colocalization panels, marks the same compartment in the inset and the surface renderings. Scale bar, 10 µm.

The phosphoinositide PI3P has been demonstrated to be a major constituent of endosomal membranes, limiting membrane of vacuoles and membranes of intraluminal vesicles of MVBs [57]. The GFP-FYVE (**F**ab1, **Y**OTB, **V**ac1 and **E**EA1) probe has been extensively used to mark the PI3P rich structures [58]. Examination of the cellular distribution of GFP-FYVE and Rgs1-mC marked structures revealed a high degree of co-localization, as determined by correlation coefficient (Pearson's coefficient = 0.34) and line scan analyses, suggesting a likely endosomal origin for Rgs1-containing vesicles and tubules (Figure 4A; Figure S2A and B in Text S1). Rgs1-mC structures were not related to filamentous mitochondria or vacuoles as judged by lack of colocalization between Rgs1-mC and mito-GFP, or Rgs1-mC and CMAC (7-amino-4-chloromethylcoumarin, a widely used membrane permeable dye that selectively stains fungal vacuoles, [59], [60], [61], [62], [63], respectively (Figure S4 B and C in Text S1).

The PI3 kinase, Vps34, facilitates the synthesis of PI3P on endosomal membranes [64]. We disrupted Vps34 function, using the specific inhibitor LY294002 [65], [66], to determine if a persistent supply of PI3P was necessary to maintain the integrity/dynamic nature of Rgs1 compartments. In contrast to the solvent (DMSO) control, which displayed extensive dynamic tubulo-vesicular structures at the hooking stage, the LY294002-treated samples were devoid of such Rgs1-mC structures. Instead, there appeared to be an accumulation of Rgs1 protein in the plasma membrane of the developing appressorium as well as vesicular aggregates in the terminal cell of the conidium (Figure 4B). Additionally, we confirmed the specificity of LY294002 by testing its effect on the GFP-FYVE strain. In contrast to the DMSO control, LY294002 treatment led to enlarged compartments containing an accumulation of the GFP-FYVE probe (Figure S5 in Text S1). Based on these results, we conclude that Rgs1 localizes to the P13P-rich endosomal network, in a manner dependent upon sustained PI3 kinase activity.

### Rgs1 colocalizes with Rab7 positive late endosomes

Based on the selective presence of either of the specific small GTPases, namely Rab5 or Rab7, the endosomal system is broadly classified into early and late compartments, respectively [67]. To identify the specific endosomal compartments that associate with active Gα_S_ and Rgs1, we carried out a colocalization analysis between Rgs1-mC and GFP-tagged Rab5 or Rab7 (Figure S6A and B in Text S1). Rgs1-mC clearly colocalized with Rab7 positive late endosomes (Pearson's coefficient = 0.48) (Figure 4C; Figure S2A and B in Text S1; Figure S7 in Text S1; Video S2), whereas no correlation was observed between the localization of Rgs1-mC and Rab5 marked early endosomes (Pearson's coefficient = 0.11) (Figure 4D; Figure S2A and B in Text S1).

To additionally assess whether the MagA^G187S^-GFP or Rgs1-mC harboring compartments were distinct from vacuolar compartments during the hooking stage (4 hpi), we carried out a co-staining analysis between MagA^G187S^-GFP or Rgs1-mC with the vacuolar dye CMAC. We found no colocalization between such GFP or mCherry marked compartments and vacuoles within the incipient appressorium. However, we did observe a distinct colocalization between CMAC and GFP or mCherry in the conidial cells (Figure S8A and B in Text S1). Taken together, these results clearly demonstrate that Rgs1 specifically localizes to the late endosomes. Furthermore, we propose that Rgs1 compartments, and by inference the late endosomes, likely represent sites of active G-protein signaling and/or anchoring in *M. oryzae*.

### Rgs1/late endosomal dynamics are facilitated by the microtubule cytoskeleton

In polarized cells, either the microtubule and/or the actin cytoskeleton facilitate the transport of a wide range of intracellular vesicular cargo/compartments. We determined the nature of the cytoskeletal tracks engaged by Rgs1 for mobility by treating samples at the hooking stage, with either MBC (Methyl Benzimidazol-2-yl-Carbamate, to destabilize the microtubule tracks) or latranculin A (LatA, to disrupt actin) [68], [69], [70].

**Figure 5:**
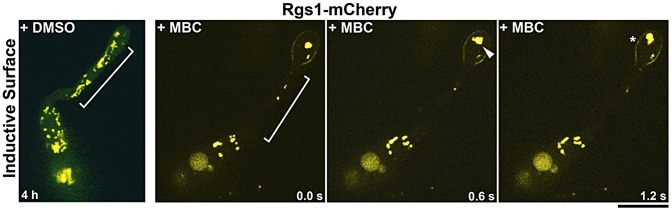
Rgs1 utilizes microtubules to traverse along the germ tube. Destabilization of the microtubule cytoskeleton disrupts Rgs1 dynamics. MBC treatment leads to Rgs1-mC (pseudocolored yellow) accumulation along the plasma membrane in the developing appressorium (asterisk) and cytoplasmic aggregation or clustering (arrowhead). The region of the germ tube lacking the Rgs1-mC tubulo-vesicular structures is bracketed. DMSO-treated sample served as a negative control. Elapsed time (in seconds) is recorded in panels D and E. Scale bar, 10 µm.

Compared to the DMSO control, the dynamics and morphology of Rgs1-mC tubulo-vesicular structures were completely disrupted upon treatment with MBC (Figure 5). Additionally, MBC treatment caused an accumulation of the Rgs1-mC on the plasma membrane and as intracellular aggregates confined to the incipient appressorium and the terminal cell of the conidium. As expected, Rgs1-mC containing structures were coincident with microtubules (GFP-α Tub), supporting the microtubule-dependent trafficking of Rgs1 (Figure S9A in Text S1) in *M. oryzae*. In contrast, LatA-treated samples showed typical mobile/dynamic tubulo-vesicular Rgs1 compartments although at a slightly reduced extent compared to the control sample (Figure S9B in Text S1). Collectively, our results show that the dynamic mobility of Rgs1-containing late endosomal compartments requires an intact microtubule cytoskeleton.

### Late endosomal maturation and function is essential for proper cAMP signaling and pathogenicity in *M. oryzae*

**Figure 6:**
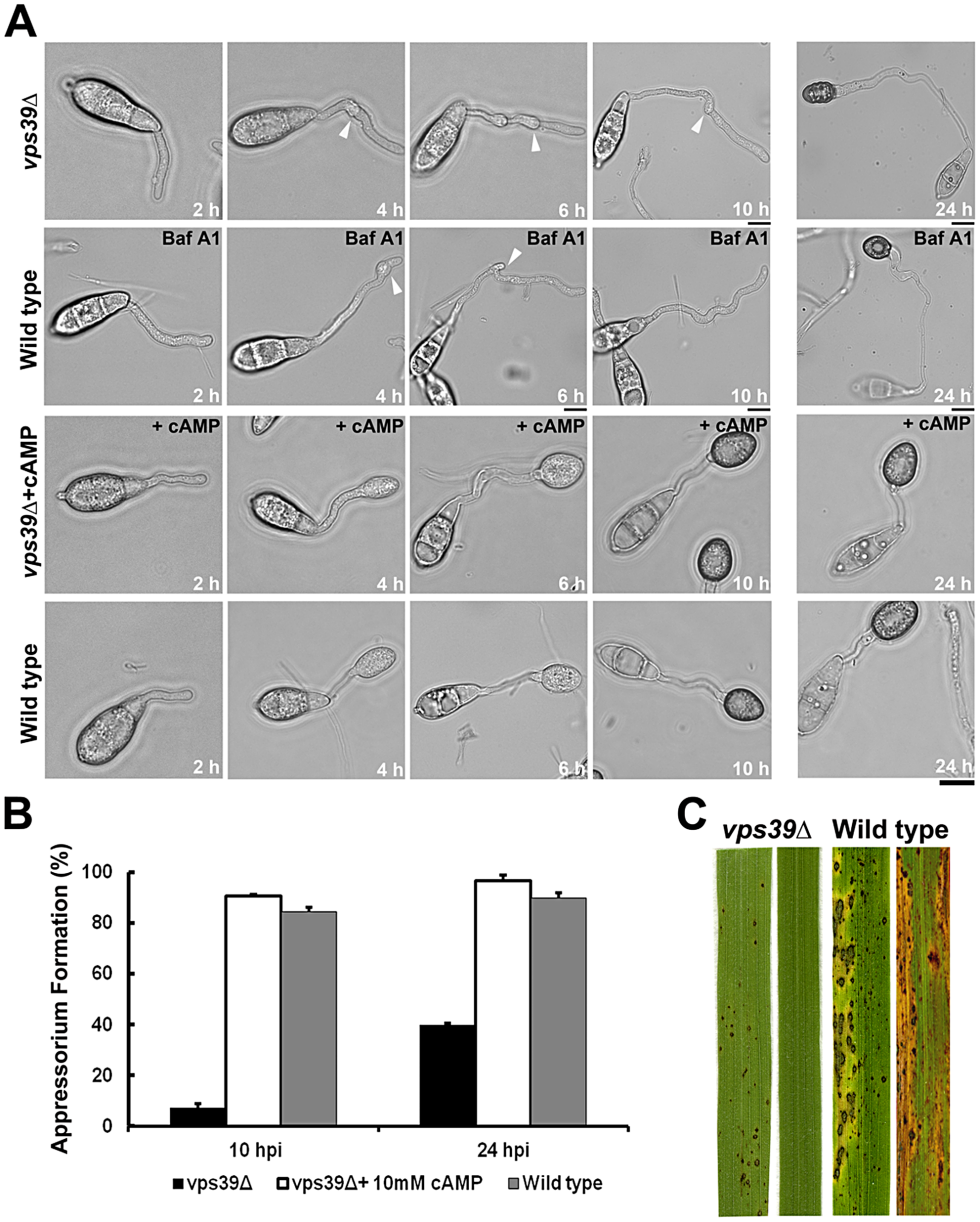
The late endosomal/HOPS component Vps39 is essential for proper cAMP signaling and pathogenesis. (**A**) Time course depicting delayed appressorium formation and multiple hooking defects (white arrowheads) in the *vps39*Δ strain on an inductive surface (row one). A *vps39*Δ-like phenotype in the wild type upon treatment with Bafilomycin A1 (Baf A1) on an inductive surface (row two). Rescue of the *vps39*Δ phenotype by exogenous cAMP (row three). Wild-type strain served as a control (row four). Images are DIC from a single slice; elapsed time (hpi) displayed. Scale bars depict 10 µm. (**B**) Bar graph illustrating the efficiency of appressorium formation in the *vps39*Δ strain. Values represent mean ± S.E from three independent replicates, 300 conidia per sample. (**C**) Blast infection assays. Conidia from the indicated strains were inoculated on the susceptible rice (CO39) seedlings and imaged 14 d post inoculation.

Vps39 (Vacuolar Protein Sorting 39), a key member of the HOPS (homotypic fusion and vacuole protein sorting) complex, plays a crucial role in the conversion of early endosomes into late endosomal compartments. Disruption of Vps39 function blocks endosomal conversion, producing merged organelles with characteristics of both early and late endosomes [71]. Our results so far support a model in which the late endosomes function as a likely scaffolding platform for active G-protein signaling. To verify our hypothesis and to understand the physiological relevance of late endosomes in cAMP signaling, we disrupted the *VPS39* function in *M. oryzae*. The *vps39*Δ mutant showed significant defects in appressorium initiation, formation and maturation, when compared to the wild-type strain at identical time points (Figure 6A). The *vps39*Δ conidia failed to form proper appressoria even upon prolonged incubation on inductive surface (24 h; Figure 6A). On an inductive surface, the *vps39*Δ germ tubes showed likely surface sensing and/or signaling defects (multiple hooking but failed appressorium formation), indicative of a lack of proper activation and regulation of cAMP signaling. In addition, disruption of endosomal acidification and hence its function and organization using bafilomycin A1 (an inhibitor of V-ATPases) [72] resulted in a *vps39*Δ like phenotypic defect in the wild type *M. oryzae* (Figure 6A). Remarkably, exogenous addition of 8-Br-cAMP completely suppressed such surface sensing and appressorium formation defects in the *vps39*Δ (Figure 6A).

The untreated *vps39*Δ strain failed to form proper appressoria even at 24 hpi, and instead showed aberrantly long germ tubes. Only 5% and 40% of *vps39*Δ conidia formed immature appressoria by 10 and 24 hpi, respectively (Figure 6B). We carried out infection assays on barley as well as a susceptible rice cultivar (CO39) to test the ability of the aberrant *vps39*Δ appressoria to cause blast disease. Disease symptoms were assessed seven and fourteen days post inoculation in barley and rice, respectively. Compared to the wild type, which caused blast disease with characteristic spindle shaped lesions with gray centers, the *vps39*Δ failed to cause disease or at best elicited only a mild hypersensitive response on rice seedlings (Figure 6C) and barley leaf explants (Figure S10B in Text S1). Furthermore, the *vps39*Δ strain was additionally compromised for radial as well as aerial growth (Figure S10A in Text S1). The number of conidia produced by the mutant was nearly ∼2.5 fold lesser compared to the wild type. Only 10% of the *vps39*Δ appressoria were functional or were successfully able to penetrate the host tissue (Figure S10C in Text S1). In conclusion, anchoring of cAMP signaling via Vps39 activity, and by inference the late endosomal/endolysosomal/HOPS function, is essential for timely and robust cAMP signaling. Additionally, late endosomal function/HOPS is necessary for pathogenesis and disease progression in *M. oryzae*.

### Adenylate cyclase localization is disrupted in the *vps39*Δ strain

**Figure 7:**
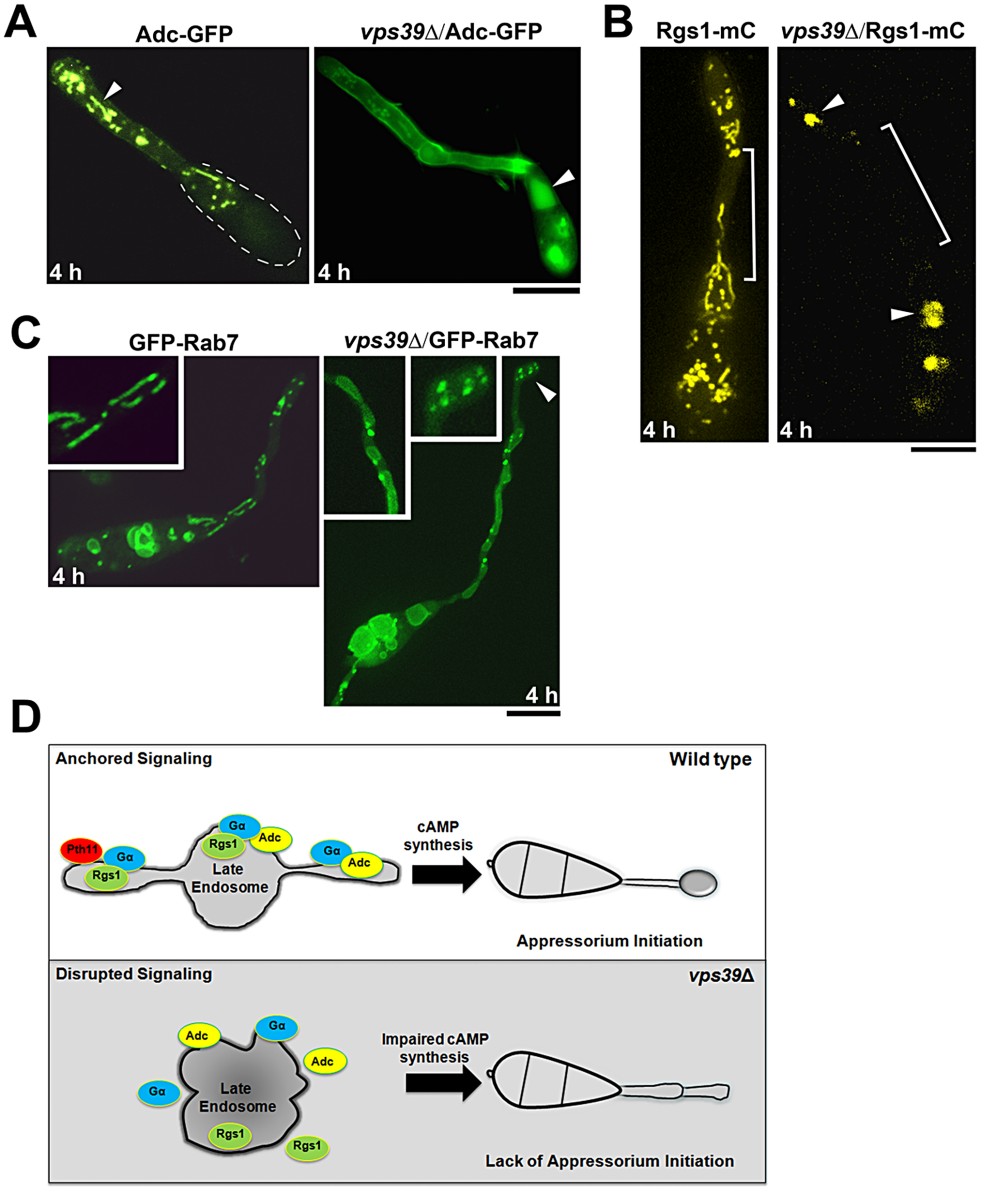
The Late Endosomal scaffolding function is necessary for proper cAMP signaling. Subcellular localization of Adenylate cyclase Mac1 (Adc-GFP), Rgs1-mCherry (mC, pseudo colored yellow) and GFP-Rab7 in the wild type and *vps39*Δ at germ tube hooking. (**A**) Adenylate cyclase Mac1 (Adc-GFP), arrowhead illustrates the localization of Adc-GFP to tubular cytosolic compartments in the wild type. The dotted line marks the boundary of the conidium (**B**) Rgs1-mCherry (mC, pseudo colored yellow); region devoid of Rgs1 tubulo-vesicular structures is bracketed and the arrowheads highlight cytoplasmic aggregates and vacuolar accumulation of Rgs1-mC in the *vps39*Δ. (**C**) GFP-Rab7; Inset highlights the morphology of GFP-Rab7 compartments in the wild type and *vps39*Δ backgrounds. All images are single plane confocal images. Scale bars, 10 µm. (**D**) A simplified model of late endosomal compartments functioning as signaling scaffolds that anchor key activators and regulators of G-protein signaling in the rice-blast fungus *M. oryzae*. This model does exclude the possible contribution of G-protein signaling/signal initiation occurring at the plasma membrane and also the possibility that signaling components are actively trafficked to the vacuole either for sequestration or for degradation to maintain cellular homeostasis.

The suppression of *vps39*Δ defects by exogenous cAMP (Figure 6A) implies that the mutant is likely impaired for cAMP synthesis and furthermore compromised for Adenylate cyclase function. We therefore assessed the subcellular localization of Adenylate cyclase (Mac1 or Adc) in the wild type and the *vps39*Δ strain at 4 hpi. Disparate from the tubulo-vesicular localization in the wild type, Mac1-GFP was predominantly cytoplasmic and/or vacuolar in the *vps39*Δ mutant (Figure 7A). Likewise, the subcellular localization of Rgs1-mC, as well as GFP-Rab7, was compromised in the *vps39*Δ strain. Both the proteins localized as intracellular aggregates or accumulated in vacuoles present in the conidia (Figure 7B and 7C).

Collectively, our results clearly support a model in which the late endosomes/endolysosomes act as anchors or platforms facilitating proper cAMP signaling. We conclude that disruption of late endosomal integrity or stability results in breakdown of cAMP synthesis and/or regulation, and leads to defects in cell signaling necessary for appressorium initiation during *M. oryzae* pathogenesis.

## Discussion

Cell shape and the spatial segregation of signaling components therein collectively influence how such molecules interact to produce a timely cellular response. Spatial separation of interacting molecules, by localization to different subcellular compartments/organelles, is a widespread mechanism of regulating pathway activity [38], [73]. Such a mechanism has been found to widely operate in mammalian cells, wherein signal compartmentalization is established via the anchoring of key molecules on scaffolding proteins called AKAPs.

In this study, we demonstrate that in the absence of authentic AKAP–like anchors [74], *M. oryzae* utilizes the dynamic late endosomal system as a common platform to anchor, transmit and directly regulate G-protein signaling during early stages of infection-related development.

Upon conidial germination on an inductive surface, the germ tube tip switches from polarized to isotropic growth (3–4 hpi). In such a context of altering cell shape, the initial pool of less mobile Rgs1 structures transform into highly dynamic tubulo-vesicular compartments. We infer that such a change is likely in response to inductive host cues and successful activation of G proteins. The ability of the late endosomes to traverse the length of the germ tube and navigate in both directions likely facilitates the recycling and/or the transport of signaling components to or away from the sites of active signaling on the plasma membrane. However, the physical interaction and preferential localization of active MagA on Rgs1-containing dynamic vesicles, suggests that the late endosomes function as ancillary sites (in addition to the plasma membrane) of active G-protein signaling in the cytosol, facilitating signal transmission through the complex cytoplasmic milieu.

We have previously demonstrated that cAMP levels in *M. oryzae* are regulated in two distinct phases, i.e., upregulated during appressorium initiation and down regulated during host invasion [53]. In this context, anchoring on dynamic endosomal structures likely provides *M. oryzae* with the flexibility and the means to rapidly assemble G-protein signaling hotspots/complexes within defined regions of the cell, namely the tip of the germ tube or within the incipient appressorium; thus gaining the ability to compartmentalize and regulate the intensity and/or duration of cAMP signaling. Furthermore, given the inherent connection(s) of the endosomal network to the vacuolar pathway, such signaling hotspots may also be rapidly disassembled to down-regulate signaling by targeting such complexes to the vacuoles, either for sequestration or degradation.

Based on the physical interaction with Rgs1 during early stages of appressorium initiation, we implicate Pth11 to be a *bona fide* GPCR (albeit non-canonical) for cAMP signaling in *M. oryzae*. Furthermore, based on the BiFC experiment, we propose that the interaction between Pth11 and Rgs1 likely occurs on the late endosomes, in the context of and in close proximity to the active MagA. It remains to be seen whether such an association requires post-translational modifications as has been demonstrated for the orthologous Sst2 (GAP) and Ste2 (receptor) in budding yeast during the mating response [75]. Incidentally, treatment with either MBC or the PI3Kinase inhibitor caused increased accumulation of Rgs1 on the plasma membrane of the incipient appressorium, suggesting that the trafficking of Rgs1 and by inference MagA likely initiates at the outer membrane upon receptor (Pth11) activation.

We found that a sustained supply of PI3P is necessary to maintain the integrity and dynamics of the G-proteins anchored on late endosomes. We have recently shown that the N-terminal DEP domain of Rgs1 facilitates specific targeting to such vesicular compartments [76]. Although we have not shown a direct interaction between Rgs1 and PI3P, a previous report has shown that the DEP domain of yeast Sst2 has the propensity to bind specific phospholipids *in vitro* [75].

Disruption of Vps39, a member of the HOPS complex, resulted in complete deregulation of surface signaling and appressorium formation in *M. oryzae*, suggesting that the late endosomal/endolysosomal function and integrity is crucial for proper cAMP signaling in *M. oryzae*. In *vps39*Δ, a significant proportion of Mac1-GFP (Adenylate cyclase) as well as Rgs1-mC localized to the vacuole. This implies that an intact late endosomal HOPS function prevents key inducers of G-protein signaling from being rapidly targeted to the vacuole for degradation during appressorium initiation. However, Rgs1-mC was predominantly vacuolar during the late stages of pathogenic growth (24 hpi) or *in planta* development (36 hpi) (Figure S11A and B in Text S1), supporting our previous finding that cAMP signaling is down regulated during the late stages of pathogenic development, thus necessitating targeting of signaling proteins to the vacuole for either sequestration, recycling and/or degradation.

The lack of proper appressorium initiation or an extensive delay in appressorium formation in the *vps39*Δ suggests that residual adenylate cyclase activity or G-protein activation likely occurs at other cellular locations, such as the plasma membrane. Such residual activity is likely insufficient to allow proper cAMP synthesis and appressorium formation, implying that the anchoring of G-proteins to the late endosomes allows sustained and robust activation of cAMP signaling in *M. oryzae*. In addition to its essential function in proper appressorium formation (and cAMP signaling) demonstrated here, we believe that endosomal fusion and maturation (Vps39 function) also contributes significantly to basic cellular functions such as proper colony growth, aerial hyphal development and conidiation (Figure S10 in Text S1).

Our results add to a growing list of recent studies that implicate endosomes, and especially the late endosomes/MVB compartments as signaling scaffolds. In *S. cerevisiae*, the constitutive active G-alpha (Gpa1^Q323L^) interacts with PI3kinase on endosomes that also contain Snf7 [64]. In mammalian cells, the MVBs play a positive role in activating Wnt signaling, by sequestering the inhibitor (GSK3) within the ILVs [44]. In another example, the free RIα subunit of cPKA is sequestered onto the surface of MVB's by AKAP11 in a cAMP dependent manner [42]. Finally, the integrity of the late endosomes is crucial for amino-acid and insulin stimulated mTOR signaling [43].

Our study revealed that dynamic tubulo-vesicular endosomes anchor active G-protein/cAMP signaling during appressorium initiation, whereas such signaling moieties are trafficked to the vacuole at late stages of infection-related morphogenesis (Figure S11A in Text S1). It was recently demonstrated that an Adenylate cyclase associated protein, Cap1, localized as bright spots in the cytoplasm of the infection hyphae in *M. oryzae* [77]. However, we could not detect such punctate localization pattern for Rgs1-mC during infectious growth. Rgs1-mC was predominantly vacuolar in the infection hyphae (Figure S11B in Text S1). The detailed localization and function (if any) of G-protein/cAMP signaling *in planta* would form the basis of future research in blast disease.

In conclusion, our findings clearly suggest that in addition to the canonical role(s), the late endosomal compartments function as legitimate anchors (Figure 7D) that integrate trafficking and signal transduction for G-proteins and cAMP synthesis during pathogenesis in the devastating rice blast fungus.

## Materials and Methods

### Fungal strains and culture conditions

The *M. oryzae* wild-type strain B157 was obtained from the Directorate of Rice Research (Hyderabad, India). For growth and conidiation, wild type and mutant strains were grown on prune agar medium (PA; per L: 40 mL prune juice, 5 g lactose, 5 g sucrose, 1 g yeast extract and 20 g agar, pH 6.5) as described [53]. Mycelial plugs were subcultured onto PA plates and incubated in a 28°C incubator in the dark for 7 days. For conidiation, cultures was incubated at 28°C in the dark for two days followed by light induction by exposing the plates to continuous fluorescent light at room temperature for 7 days. All the tagged strains used in this study were characterized for defects in vegetative growth, conidiation, appressorium formation, ability to respond to inductive/non-inductive surfaces, and pathogenicity (Figure S12 and 13 in Text S1). The wild-type strain was used as a control. The strains were also evaluated for protein expression and the size of the GFP- or mCherry fusion proteins was verified using western blot analysis (Figure S12 and 13 in Text S1). All the strains were found to be wild type like and the tagged proteins were found to be biologically functional. Experimental results were verified with a minimum of two strains of the same genotype. All strains used in this study were validated by Southern blotting, locus specific PCRs and requisite DNA sequence analyses. We have carefully compared and ascertained that constitutive promoter driven expression does not compromise the function, localization, and dynamics of the tagged Pth11 or Rgs1 compared to the natively expressed GFP-tagged versions. Only the intensity of the signal was enhanced thus allowing for better live-cell imaging. In the MagA^G187S^-GFP, MagA-GFP, Mac1-GFP, Rgs1-GFP and Pth11-GFP strains, the fusion protein in each instance is the sole copy of the given protein in that strain.

### Evaluation of pathogenicity

For pathogenicity assays, leaves from two-week old barley seedlings were cut into small pieces (2–3 cm long) and washed in sterile water, following which the leaf explants were rinsed for 45 seconds in 40% ethanol. The leaf pieces were then washed twice with sterile antibiotic containing distilled water. The washed leaves were placed on kinetin agar medium (2 mg/mL kinetin, 1% agar). Conidia were quantified and a dilution series of the conidial suspension was inoculated on detached barley leaves at the required concentrations. The samples were incubated in a humidified growth chamber with a 16 h light/8 h dark cycle at 22°C. Disease symptoms were assessed 5–7 days post inoculation.

### Plasmid constructs for the expression of *RGS1*-mCherry fusion

The *RGS1* gene was tagged with mCherry at the C-terminus since the N-terminal DEP-DEP domain is known to specify membrane targeting [76]. The mCherry ORF was PCR amplified from pmCherry (BD Biosciences Clontech, USA). The *RGS1* gene (MGG_14517) was amplified from genomic DNA of wild type *M. oryzae*. The TrpC terminator was amplified using pFGL275 as template. The PCR products were digested with appropriate restriction enzymes (New England Biolabs, Beverly, MA) and purified using the gel elution Nucleospin Extract II kit (Machery-Nagel, Easton, PA). Using a three way ligation approach the mCherry (*Sma*I/*BamH*I) fragment along with the TrpC terminator as a *BamH*I/*Xba*I fragment were cloned into the *Sma*I/*Xba*I sites of pFGL44 vector [encoding hygromycin phosphotransferase gene (*HPH1*)] to obtain pFGL44-mCherry-TrpC terminator. The digested and eluted *RGS*1 (*EcoR*I/*Sma*I fragment) and Ccg1 (*EcoR*I-*EcoR*I) fragments were sequentially cloned into pFGL44-mCherry-TrpC, to yield pFGL44-*RGS*1-mCherry-TrpC and subsequently to give pFGL44-Ccg1-*RGS*1-mCherry-TrpC, representing the final construct. The orientation of the Ccg1 promoter fragment in the final constructs was confirmed using *Hind*III and *Kpn*I restriction enzymes. The final clones were subjected to sequence analysis and then introduced into the wild type B157 strain via *Agrobacterium* T-DNA-mediated transformation. Fungal transformants were selected based on resistance towards hygromycin (CM containing 250 µg/ml hygromycin, A.G. Scientific Inc, USA). Fungal transformants were screened for Rgs1-mCherry expression using an epifluorescence microscope and confirmed by sequencing of the requisite genomic region.

### Construction of plasmid vector for eGFP-α Tubulin

The α-Tubulin gene was tagged at the N-terminus with eGFP driven by the RP27 promoter [55], [78]. The eGFP was PCR amplified from pEGFP-N1 (BD Biosciences Clontech, USA). The RP27 promoter and the α-tubulin gene (MGG_06650) were amplified using genomic DNA from extracted from wild type *M. oryzae* as template. Plasmid pFGL275 was used as a template for the amplification of the TrpC terminator. The PCR products obtained were digested with suitable restriction enzymes (New England Biolabs, Beverly, MA) and purified using the gel elution Nucleospin Extract II kit (Machery-Nagel, Easton, PA). The TrpC terminator as a *Sma*I/*BamH*I fragment was cloned into the *Sma*I/*BamH*I sites of pFGL97 vector [encoding ammonium glufosinate gene (*BAR*)] to obtain pFGL97- TrpC terminator. The digested and eluted eGFP (*Nco*I/*Sma*I fragment) and RP27 (*EcoR*I/*Nco*I) fragments were cloned using a three way ligation approach into pFGL97-TrpC, to yield pFGL97-RP27-eGFP-TrpC. Finally the α-tubulin was cloned as a *Sma*I/*Sma*I fragment into pFGL97-RP27-eGFP-TrpC to subsequently yield pFGL97-RP27-α-tubulin-eGFP-TrpC, representing the final construct. The orientation of the α-tubulin fragment in the final construct was confirmed using *BamH*I restriction enzyme. The final clones were subjected to sequence analysis and were then introduced into the appropriate background (Rgs1-mCherry) strain via *Agrobacterium* T-DNA-mediated transformation. Fungal transformants were initially selected using ammonium glufosinate resistance (BM containing 50 µg/ml ammonium glufosinate, Cluzeau Info Labo, France) and confirmed by genomic DNA sequencing and GFP expression.

### Construction of plasmid vector for eGFP-Vps21/Rab5 and eGFP-Ypt7/Rab7

The Rab5 ortholog in *M. oryzae*, Vps21 gene (MGG_06241) was also tagged at the N-terminus with eGFP and driven by the RP27 promoter [55], [78]. C-terminal tagging of Vps21/Rab5 is known to disrupt protein function. The eGFP was PCR amplified from pEGFP-N1 (BD Biosciences Clontech, USA). The RP27 promoter and the Vps21 gene were amplified using genomic DNA from extracted from wild type as template. Plasmid pFGL275 was used as a template to amplify the TrpC terminator. The PCR products obtained were digested with suitable restriction enzymes (New England Biolabs, Beverly, MA) and purified using the gel elution Nucleospin Extract II kit (Machery-Nagel, Easton, PA). First, the digested Vps21 gene, digested with *Sma*I/*BamH*I was cloned into pFGL97 vector [encoding ammonium glufosinate gene (*BAR*)] at the *Sma*I/*BamH*I sites, followed by the TrpC terminator as a *BamH*I/*BamH*I fragment to obtain pFGL97-Vps21-TrpC terminator. The orientation of the TrpC terminator was confirmed using *Nco*I/*Pst*I restriction enzymes. Next, the digested and eluted RP27 (*EcoR*I/*Nco*I) and eGFP (*Nco*I/*Sma*I fragment) fragments were cloned using a three way ligation approach into pFGL97-Vps21-TrpC, to yield pFGL97-RP27-eGFP-Vps21-TrpC, the final construct.

The Rab7 ortholog Ypt7 (MGG_08144) in *M. oryzae* was also tagged at the N-terminus, utilizing the pFGL97-RP27-eGFP-TrpC vector as the base for cloning. As mentioned above, the Ypt7 gene was PCR amplified using wild type genomic DNA as template. The Ypt7 amplicon was digested with *Sma*I enzyme and cloned into the pFGL97-RP27-eGFP-TrpC vector, at the *Sma*I site. This yielded the final construct pFGL97-RP27-eGFP-Ypt7-TrpC. The orientation of cloned Ypt7 fragment was checked and confirmed by digestion with *Hind*III.

In both the cases, the final clones were subjected to sequence analysis and were then introduced into the appropriate background strains (*RGS1*-mCherry) via *Agrobacterium* T-DNA-mediated transformation. Requisite transformants were initially screened by epifluorescence microscopy for GFP expression, and candidate strains confirmed by locus-specific PCR and genomic DNA sequencing.

### Construction of plasmid vector for eGFP-2X FYVE

The eGFP-2X FYVE fragment was released from pEGFP-2X FYVE (a kind gift from Prof Harald Stenmark) as an *Nco*I-*Sma*I fragment. This fragment (*Nco*I-*Sma*I), along with the RP27 promoter (*EcoR*I-*Nco*I) was cloned into pFGL97 vector using a three-way ligation strategy to yield pFGL97-RP27-eGFP-2X FYVE. The TrpC terminator was cloned next into the same vector as *Sma*I/*BamH*I fragments to yield the final construct pFGL97-RP27-eGFP-2X FYVE-TrpC. Transformants were identified and confirmed as described above.

### Construction of plasmid vector for active Gα_S_-GFP (MagA^G187S^-GFP) and wild type Gα_S_-GFP (MagA-GFP)

Tagging at the C- or the N-terminus with fluorescent marker is known to compromise the function of the G-alpha subunit. In order to circumvent this problem, we introduced the eGFP coding sequence in frame into the alphaB-alphaC loop of *M. oryzae* MagA/Gαs (between amino acids 113–120; as suggested by D. Siderovski and D. Bosch, USA based on an earlier study [56]. Based on an alternate strategy in *D. discoideum* [79], the Gα subunits in *N. crassa* were recently tagged with TagRFP in the first alphaA-alphaB loop [80]. We used vector pFGL718 in which the fragment encoding ammonium glufosinate and eGFP is available as an *Hpa*I-*Mlu*I fragment. Genomic DNA from the wild type was used as template to amplify the regions flanking either side of the alphaB-alphaC loop region in the MagA protein. The upstream flank was amplified as an *EcoR*I-*Mlu*I fragment and the downstream flank as an *Hpa*I-*Hind*III fragment. Both the flanks were amplified such that they also incorporated short six amino acid linkers. In addition, another downstream flank incorporating the G187S point mutation was also amplified as an *Hpa*I -*Hind*III fragment, using genomic DNA from the MagA^G187S^ strain [54]. The sequence of the *Hpa*I-*Hind*III fragment incorporating the G187S point mutation was sequenced and verified. The single 5′ upstream fragment and the two 3′ flanks were digested with appropriate restriction enzymes and cloned into pFGL718, such that the upstream flank, the *BAR*-eGFP fragment and the downstream flank were all in frame. The constructs were confirmed and integrated into the Rgs1-mCherry strain such that the GFP-tagged MagA replaced the wild-type copy by homologous recombination. Site-specific replacement was confirmed using Southern analysis, locus-specific PCR and nucleotide sequencing.

### Construction of plasmid vectors for BiFC strain expressing (RP27-Pth11-YFP^1–156^) and (Ccg1-Rgs1-YFP^156–239^)

A BiFC strain expressing Pth11-YFP^1–156^ and Rgs1-YFP^156–239^ was generated to study at the interactions between the two proteins *in-vivo*. For this purpose, two constructs were generated, one in which *PTH11* was cloned into pFGL44 and tagged C-terminally to YFP^1–156^ under the RP27 promoter; whereas the second construct involved cloning *RGS1* into pFGL97 and tagged at the C-terminus to YFP^156–239^ under the Ccg1 promoter. The selected strains were confirmed by PCR analysis with appropriate controls.

### Construction of *vps39*Δ strain

The *VPS39* deletion mutant was generated using the standard one-step gene replacement strategy. Genomic DNA fragments (about 1 kb each) representing the 5′ and 3′ UTR of *VPS39* (MGG_01054) gene were amplified by PCR, ligated sequentially so as to flank the ammonium-glufosinate resistance gene cassette (*BAR*) under the TrpC promoter in pFGL97 to obtain plasmid vector pFGLvps39-KO. The sequence of pFGLvps39-KO gene replacement constructs was confirmed and introduced into Rgs1-mCherry, GFP-Rab7 and Mac1-GFP strains via Agrobacterium T-DNA-mediated transformation to specifically replace the *VPS39* with *BAR* resistance cassettes. Resistance to ammonium glufosinate (BM containing 40 ug/ml ammonium glufosinate (Cluzeau Labo, France) was used to select transformants.

### Immuno-precipitation assay utilizing GFP and RFP-Trap

Total protein was extracted using non-denaturing NP40 buffer (10 mM Tris/Cl, pH 7.5, 150 mM NaCl, 0.5 mM EDTA, 0.5% NP40, 1 mM PMSF and 1× Protease Inhibitor Cocktail). Total protein was extracted from conidia inoculated on an inductive surface at the hooking stage (4 hpi) or from vegetative growth from the requisite strains. Equal concentration of total protein lysate was incubated with GFP/RFP-Trap agarose beads (ChromoTek, Germany) and immuno-precipitation carried out as per manufacturer's instructions. Immuno-precipitated samples was fractionated by SDS–PAGE (on 4–20% pre-cast gels, Bio-Rad, USA), transferred onto a PVDF membrane (Millipore Corporation, USA) and immuno blotted with α-Rgs1 (DEP-DEP domain specific) , α-GFP or α-mCherry antiserum (1∶1000 dilution). Secondary antibodies conjugated to horseradish peroxidase were used at 1∶10000 dilutions. The Super Signal kit (Pierce, USA) was used to detect the chemi-luminescent signal as instructed [54], [76].

### Appressorial assays, chemical inhibitors and rescue experiments

For appressorial assays and imaging using bright field optics, harvested conidia were resuspended in sterile water at a required concentration. Droplets (20–40 µl) containing conidial suspension was placed on hydrophobic plastic coverslips or glass bottom dishes (Mat Tek Corporation, USA) and incubated under humid conditions at room temperature. The Rgs1-mC strain was inoculated on hydrophobic plastic coverslips and treated for 30 minutes with MBC (final concentration 1 µM) and LatA (final concentration 10 µM) at the hooking stage. PI3Kinase inhibitor LY294002 was added to Rgs1-mCherry conidia germinated on an inductive surface 4 hpi at a final concentration of 100 µM. The treatment was carried out for 180 minutes. In the case of *GFP-FYVE* strain, the LY294002 treatment was also carried out 4 hpi on an inductive surface, for 60–90 minutes. Rescue of the *vps39*Δ mutant was carried out by adding 8-Br-cAMP to a final concentration of 10 mM at 0 hpi. Wild type was treated with Bafilomycin-A1 2 hpi at a final concentration of 50 nM. A 0.1% DMSO solvent control was used in all assays. CMAC was used at a final concentration of 10 µM. Staining with CMAC was usually carried out 3–4 hpi, on an inductive surface, for 10–15 min at 37°C. The sample was washed and subsequently visualized. Lysotracker Green was used at a final concentration of 50 nM. Staining was carried out for 10 min at 37°C, 4 hpi, on an inductive surface.

### Live cell imaging and colocalization analysis

Droplets (20–40 µl) containing conidial suspension were placed on hydrophobic plastic coverslips or glass bottom dishes (Mat Tek Corporation, USA) and incubated in a moist chamber at room temperature. Imaging was performed at the specified time points. Bright field images were captured at desired time points post inoculation using an Olympus BX51 microscope equipped with a PlanAPO 100×/1.45 or UPlan FLN 60×/1.25 objective with appropriate filter sets. Images were captured using a CoolSNAP HQ camera (Photometrics, USA) and processed using MetaVue (Universal Imaging, USA).

Time-lapse or live cell fluorescence microscopy was performed using a Zeiss Axiovert 200 M microscope (Plan Apochromat 100×, 1.4NA objective) equipped with an UltraView RS-3 spinning disk confocal system (PerkinElmer Inc., USA) using a CSU21 confocal optical scanner, 12-bit digital cooled Hamamatsu Orca-ER camera (OPELCO, Sterling, VA, USA) and a 491 nm 100 mW and a 561 nm 50 mW laser illumination under the control of MetaMorph Premier Software, (Universal Imaging, USA). Typically, z-stacks consisted of 0.5 µm-spaced planes for every time point. The maximum projection was obtained using the Metamorph built-in module. Alternatively, images were acquired using a Nikon TiE system (CFI Plan Apochromat VC 100XH 1.4 N.A. objective) equipped with a Yokogawa CSU-X1-A1 spinning disk unit, a Photometrics CoolSNAP HQ2 camera and a DPSS 491 nm 100 mW and DPSS561 nm 50 mW laser lines under the control of MetaMorph Premier Software, (Universal Imaging, USA). Typically, single z-plane or image stacks that consisted of 0.5 µm-spaced sections were captured. GFP and LTG excitation were performed at 491 nm (Em. 525/40 nm), FM4-64 excitation at 491 nm (Em. 607/36 nm), mCherry excitation at 561 nm (Em. 607/36 nm), YFP excitation at 515 nm (Em. 542/27 nm) and CMAC excitation at 405 nm (Em. 452/45 nm).

For quantitation of colocalization the raw .STK files were opened as stacks in Imaris, and the extent of colocalization between Rgs1-mCherry and the other GFP-tagged proteins or endosomal markers was measured using the Colocalization module of Imaris 7.5, 64-bit version (Bitplane AG, Saint Paul, MN, www.bitplane.com). Two-channel Z-stacks were assembled and the relative intensity of each wavelength determined for each voxel. The minimum intensity threshold for the analysis was determined for each channel automatically using the iterative algorithm defined by Costes et al. [81]. A time-dependent analysis of colocalization was run for each sample. Both the percentage of material colocalized (as determined by the number of colocalized voxels relative to the total number of voxels with an intensity value of GFP signal above the minimum intensity threshold) and the Pearson's colocalization coefficient was calculated from each sample for a minimum of four time points, a minimum of three different cells and at least two independent experiments performed on different days. The data were pooled and the results represent the average with error bars corresponding to the standard error of the mean.

### Image processing

Image processing and figure preparation was performed using Bitplane Imaris for 3D surface rendering and colocalization analysis (see above), Fiji (http://fiji.sc/wiki/index.php/Fiji), and Adobe Photoshop and Microsoft Excel (for figure preparation).

### GenBank accession numbers

Sequences for genes described in this article can be found under the following accession numbers: *M. oryzae RGS1* (MGG_14517), *MAG A* (MGG_01818), *PTH11* (MGG_05871), *VPS21* (MGG_06241), *YPT7* (MGG_08144), *TUB* (MGG_06650), *VPS39* (MGG_01054) and *MAC1* (MGG_09898)

## Supporting Information

Text S1 Supporting Figures and Tables: Details about (a) Dynamics of Rgs1-mCherry during growth on a non-inductive surface and in the *pth11*Δ strain (b) Pearson's coefficient analysis and line scan graphs for colocalization between Rgs1 and specific marker proteins (c) BiFC experiment showing *in-vivo* interaction between Pth11 and Rgs1 at 2 hpi and controls for the hooking stage (4 hpi) (d) Colocalization analysis between Rgs1 and intracellular organelles (e) Effect of PI3 kinase inhibitor LY294002 on the GFP-FYVE strain (f) Orthologs for early or late endosome specific Rab GTPases in *M. oryzae* (g) Specific colocalization between Rgs1 and the late endosomal moieties (h) Colocalization between MagA^G187S^-GFP and Rgs1-mCherry and CMAC stained vacuoles (i) Role of F-actin cytoskeleton in Rgs1 dynamics (j) Colony morphology, aerial growth and disease causing ability of the *vps39*Δ strain (k) Subcellular localization of Rgs1-mC (24 hpi) and during *in-planta* growth (36 hpi) (l) Characterization and western blot analysis of strains used in this study (m) Characterization, quantification of conidiation and appressorial function of the strains used in this study (n) Oligonucleotide primers used in this study.
(PDF)

Video S1 Dynamics and Mobility of Rgs1. Rgs1-mCherry in tubulo-vesicular compartments, 4 hpi, on an inductive surface. Movie shows a time series of maximum intensity Z-projection of confocal stacks of five slices; elapsed time is indicated in seconds.
(MOV)

Video S2 Colocalization of Rgs1 with Late endosomes. GFP-Rab7 marked late endosomes associate with Rgs1-mCherry structures at the hooking stage. Movie shows a time series of images that are maximum intensity Z-projections of confocal stacks; elapsed time is indicated in seconds and the associated Pearson's correlation coefficient is displayed for each time point.
(MOV)

## References

[1] Dohlman HG, Thorner JW (2001) Regulation of G protein-initiated signal transduction in yeast: paradigms and principles. Annu Rev Biochem 70: 703–754. 11395421 10.1146/annurev.biochem.70.1.703

[2] Tesmer JJ, Sprang SR (1998) The structure, catalytic mechanism and regulation of adenylyl cyclase. Curr Opin Struct Biol 8: 713–719. 9914249 10.1016/s0959-440x(98)80090-0

[3] Neves SR, Ram PT, Iyengar R (2002) G protein pathways. Science 296: 1636–1639. 12040175 10.1126/science.1071550

[4] Bosch DE, Willard FS, Ramanujam R, Kimple AJ, Willard MD, et al (2012) A P-loop mutation in Galpha subunits prevents transition to the active state: implications for G-protein signaling in fungal pathogenesis. PLoS Pathog 8: e1002553. 22383884 10.1371/journal.ppat.1002553 PMC3285607

[5] Dohlman HG, Song J, Apanovitch DM, DiBello PR, Gillen KM (1998) Regulation of G protein signalling in yeast. Semin Cell Dev Biol 9: 135–141. 9599408 10.1006/scdb.1998.0218

[6] Siderovski DP, Willard FS (2005) The GAPs, GEFs, and GDIs of heterotrimeric G-protein alpha subunits. Int J Biol Sci 1: 51–66. 15951850 10.7150/ijbs.1.51 PMC1142213

[7] Berman DM, Wilkie TM, Gilman AG (1996) GAIP and RGS4 are GTPase-activating proteins for the Gi subfamily of G protein alpha subunits. Cell 86: 445–452. 8756726 10.1016/s0092-8674(00)80117-8

[8] Hunt TW, Fields TA, Casey PJ, Peralta EG (1996) RGS10 is a selective activator of G alpha i GTPase activity. Nature 383: 175–177. 8774883 10.1038/383175a0

[9] Lan KL, Sarvazyan NA, Taussig R, Mackenzie RG, DiBello PR, et al (1998) A point mutation in Galphao and Galphai1 blocks interaction with regulator of G protein signaling proteins. J Biol Chem 273: 12794–12797. 9582306 10.1074/jbc.273.21.12794

[10] Tesmer JJ, Berman DM, Gilman AG, Sprang SR (1997) Structure of RGS4 bound to AlF4–activated G(i alpha1): stabilization of the transition state for GTP hydrolysis. Cell 89: 251–261. 9108480 10.1016/s0092-8674(00)80204-4

[11] Watson N, Linder ME, Druey KM, Kehrl JH, Blumer KJ (1996) RGS family members: GTPase-activating proteins for heterotrimeric G-protein alpha-subunits. Nature 383: 172–175. 8774882 10.1038/383172a0

[12] Li L, Wright SJ, Krystofova S, Park G, Borkovich KA (2007) Heterotrimeric G protein signaling in filamentous fungi. Annu Rev Microbiol 61: 423–452. 17506673 10.1146/annurev.micro.61.080706.093432

[13] Lemaire K, Van de Velde S, Van Dijck P, Thevelein JM (2004) Glucose and sucrose act as agonist and mannose as antagonist ligands of the G protein-coupled receptor Gpr1 in the yeast Saccharomyces cerevisiae. Mol Cell 16: 293–299. 15494315 10.1016/j.molcel.2004.10.004

[14] Maidan MM, De Rop L, Serneels J, Exler S, Rupp S, et al (2005) The G protein-coupled receptor Gpr1 and the Galpha protein Gpa2 act through the cAMP-protein kinase A pathway to induce morphogenesis in Candida albicans. Mol Biol Cell 16: 1971–1986. 15673611 10.1091/mbc.E04-09-0780 PMC1073676

[15] Welton RM, Hoffman CS (2000) Glucose monitoring in fission yeast via the Gpa2 galpha, the git5 Gbeta and the git3 putative glucose receptor. Genetics 156: 513–521. 11014802 10.1093/genetics/156.2.513 PMC1461262

[16] Xue C, Bahn YS, Cox GM, Heitman J (2006) G protein-coupled receptor Gpr4 senses amino acids and activates the cAMP-PKA pathway in Cryptococcus neoformans. Mol Biol Cell 17: 667–679. 16291861 10.1091/mbc.E05-07-0699 PMC1356578

[17] Li L, Borkovich KA (2006) GPR-4 is a predicted G-protein-coupled receptor required for carbon source-dependent asexual growth and development in Neurospora crassa. Eukaryot Cell 5: 1287–1300. 16896213 10.1128/EC.00109-06 PMC1539153

[18] Lafon A, Seo JA, Han KH, Yu JH, d'Enfert C (2005) The heterotrimeric G-protein GanB(alpha)-SfaD(beta)-GpgA(gamma) is a carbon source sensor involved in early cAMP-dependent germination in Aspergillus nidulans. Genetics 171: 71–80. 15944355 10.1534/genetics.105.040584 PMC1456537

[19] Doehlemann G, Berndt P, Hahn M (2006) Different signalling pathways involving a Galpha protein, cAMP and a MAP kinase control germination of Botrytis cinerea conidia. Mol Microbiol 59: 821–835. 16420354 10.1111/j.1365-2958.2005.04991.x

[20] Bardwell L (2005) A walk-through of the yeast mating pheromone response pathway. Peptides 26: 339–350. 15690603 10.1016/j.peptides.2004.10.002 PMC3017506

[21] Seo JA, Han KH, Yu JH (2005) Multiple roles of a heterotrimeric G-protein gamma-subunit in governing growth and development of Aspergillus nidulans. Genetics 171: 81–89. 15944346 10.1534/genetics.105.042796 PMC1456535

[22] Seo JA, Yu JH (2006) The phosducin-like protein PhnA is required for Gbetagamma-mediated signaling for vegetative growth, developmental control, and toxin biosynthesis in Aspergillus nidulans. Eukaryot Cell 5: 400–410. 16467480 10.1128/EC.5.2.400-410.2006 PMC1405901

[23] Liu S, Dean RA (1997) G protein alpha subunit genes control growth, development, and pathogenicity of Magnaporthe grisea. Mol Plant Microbe Interact 10: 1075–1086. 9390422 10.1094/MPMI.1997.10.9.1075

[24] Nishimura M, Park G, Xu JR (2003) The G-beta subunit MGB1 is involved in regulating multiple steps of infection-related morphogenesis in Magnaporthe grisea. Mol Microbiol 50: 231–243. 14507377 10.1046/j.1365-2958.2003.03676.x

[25] Muller P, Leibbrandt A, Teunissen H, Cubasch S, Aichinger C, et al (2004) The Gbeta-subunit-encoding gene bpp1 controls cyclic-AMP signaling in Ustilago maydis. Eukaryot Cell 3: 806–814. 15190001 10.1128/EC.3.3.806-814.2004 PMC420130

[26] Regenfelder E, Spellig T, Hartmann A, Lauenstein S, Bolker M, et al (1997) G proteins in Ustilago maydis: transmission of multiple signals? EMBO J 16: 1934–1942. 9155019 10.1093/emboj/16.8.1934 PMC1169796

[27] Alspaugh JA, Perfect JR, Heitman J (1997) Cryptococcus neoformans mating and virulence are regulated by the G-protein alpha subunit GPA1 and cAMP. Genes Dev 11: 3206–3217. 9389652 10.1101/gad.11.23.3206 PMC316752

[28] Alspaugh JA, Pukkila-Worley R, Harashima T, Cavallo LM, Funnell D, et al (2002) Adenylyl cyclase functions downstream of the Galpha protein Gpa1 and controls mating and pathogenicity of Cryptococcus neoformans. Eukaryot Cell 1: 75–84. 12455973 10.1128/EC.1.1.75-84.2002 PMC118042

[29] Wang P, Perfect JR, Heitman J (2000) The G-protein beta subunit GPB1 is required for mating and haploid fruiting in Cryptococcus neoformans. Mol Cell Biol 20: 352–362. 10594037 10.1128/mcb.20.1.352-362.2000 PMC85090

[30] Gao S, Nuss DL (1996) Distinct roles for two G protein alpha subunits in fungal virulence, morphology, and reproduction revealed by targeted gene disruption. Proc Natl Acad Sci U S A 93: 14122–14127. 11038529 10.1073/pnas.93.24.14122 PMC19504

[31] Segers GC, Nuss DL (2003) Constitutively activated Galpha negatively regulates virulence, reproduction and hydrophobin gene expression in the chestnut blight fungus Cryphonectria parasitica. Fungal Genet Biol 38: 198–208. 12620256 10.1016/s1087-1845(02)00534-0

[32] Kasahara S, Nuss DL (1997) Targeted disruption of a fungal G-protein beta subunit gene results in increased vegetative growth but reduced virulence. Mol Plant Microbe Interact 10: 984–993. 9353946 10.1094/MPMI.1997.10.8.984

[33] Liang S, Wang Z, Liu P, Li D (2006) A Gγ subunit promoter T-DNA insertion mutant—A1-412 of Magnaporthe grisea is defective in appressorium formation, penetration and pathogenicity. Chinese Science Bulletin 51: 2214–2218.

[34] Kruger J, Loubradou G, Regenfelder E, Hartmann A, Kahmann R (1998) Crosstalk between cAMP and pheromone signalling pathways in Ustilago maydis. Mol Gen Genet 260: 193–198. 9862471 10.1007/s004380050885

[35] Liebmann B, Gattung S, Jahn B, Brakhage AA (2003) cAMP signaling in Aspergillus fumigatus is involved in the regulation of the virulence gene pksP and in defense against killing by macrophages. Mol Genet Genomics 269: 420–435. 12734751 10.1007/s00438-003-0852-0

[36] Liebmann B, Muller M, Braun A, Brakhage AA (2004) The cyclic AMP-dependent protein kinase a network regulates development and virulence in Aspergillus fumigatus. Infect Immun 72: 5193–5203. 15322014 10.1128/IAI.72.9.5193-5203.2004 PMC517480

[37] Perino A, Ghigo A, Scott JD, Hirsch E (2012) Anchoring proteins as regulators of signaling pathways. Circ Res 111: 482–492. 22859670 10.1161/CIRCRESAHA.111.262899 PMC3508715

[38] Cowan AE, Moraru II, Schaff JC, Slepchenko BM, Loew LM (2012) Chapter 8 - Spatial Modeling of Cell Signaling Networks. In: Anand RA, Adam PA, editors. Methods in Cell Biology: Academic Press. pp. 195–221. 10.1016/B978-0-12-388403-9.00008-4 PMC3519356 22482950

[39] Murphy JE, Padilla BE, Hasdemir B, Cottrell GS, Bunnett NW (2009) Endosomes: a legitimate platform for the signaling train. Proc Natl Acad Sci U S A 106: 17615–17622. 19822761 10.1073/pnas.0906541106 PMC2764915

[40] Huotari J, Helenius A (2011) Endosome maturation. EMBO J 30: 3481–3500. 21878991 10.1038/emboj.2011.286 PMC3181477

[41] Von Bartheld CS, Altick AL (2011) Multivesicular bodies in neurons: distribution, protein content, and trafficking functions. Prog Neurobiol 93: 313–340. 21216273 10.1016/j.pneurobio.2011.01.003 PMC3055956

[42] Day ME, Gaietta GM, Sastri M, Koller A, Mackey MR, et al (2011) Isoform-specific targeting of PKA to multivesicular bodies. J Cell Biol 193: 347–363. 21502359 10.1083/jcb.201010034 PMC3080257

[43] Flinn RJ, Yan Y, Goswami S, Parker PJ, Backer JM (2010) The late endosome is essential for mTORC1 signaling. Mol Biol Cell 21: 833–841. 20053679 10.1091/mbc.E09-09-0756 PMC2828969

[44] Taelman VF, Dobrowolski R, Plouhinec JL, Fuentealba LC, Vorwald PP, et al (2010) Wnt signaling requires sequestration of glycogen synthase kinase 3 inside multivesicular endosomes. Cell 143: 1136–1148. 21183076 10.1016/j.cell.2010.11.034 PMC3022472

[45] Contento AL, Bassham DC (2012) Structure and function of endosomes in plant cells. J Cell Sci 125: 3511–3518. 22935651 10.1242/jcs.093559

[46] Miaczynska M, Pelkmans L, Zerial M (2004) Not just a sink: endosomes in control of signal transduction. Curr Opin Cell Biol 16: 400–406. 15261672 10.1016/j.ceb.2004.06.005

[47] Seto ES, Bellen HJ, Lloyd TE (2002) When cell biology meets development: endocytic regulation of signaling pathways. Genes Dev 16: 1314–1336. 12050111 10.1101/gad.989602

[48] Wilson RA, Talbot NJ (2009) Under pressure: investigating the biology of plant infection by Magnaporthe oryzae. Nat Rev Microbiol 7: 185–195. 19219052 10.1038/nrmicro2032

[49] Li G, Zhou X, Xu JR (2012) Genetic control of infection-related development in Magnaporthe oryzae. Curr Opin Microbiol 5 ((6)): 678–84. 10.1016/j.mib.2012.09.004 23085322

[50] Adachi K, Hamer JE (1998) Divergent cAMP signaling pathways regulate growth and pathogenesis in the rice blast fungus Magnaporthe grisea. Plant Cell 10: 1361–1374. 9707535 10.1105/tpc.10.8.1361 PMC144070

[51] Choi W, Dean RA (1997) The adenylate cyclase gene MAC1 of Magnaporthe grisea controls appressorium formation and other aspects of growth and development. Plant Cell 9: 1973–1983. 9401122 10.1105/tpc.9.11.1973 PMC157051

[52] Lee YH, Dean RA (1993) cAMP Regulates Infection Structure Formation in the Plant Pathogenic Fungus Magnaporthe grisea. Plant Cell 5: 693–700. 12271080 10.1105/tpc.5.6.693 PMC160306

[53] Ramanujam R, Naqvi NI (2010) PdeH, a high-affinity cAMP phosphodiesterase, is a key regulator of asexual and pathogenic differentiation in Magnaporthe oryzae. PLoS Pathog 6: e1000897. 20463817 10.1371/journal.ppat.1000897 PMC2865543

[54] Liu H, Suresh A, Willard FS, Siderovski DP, Lu S, et al (2007) Rgs1 regulates multiple Galpha subunits in Magnaporthe pathogenesis, asexual growth and thigmotropism. EMBO J 26: 690–700. 17255942 10.1038/sj.emboj.7601536 PMC1794393

[55] DeZwaan TM, Carroll AM, Valent B, Sweigard JA (1999) Magnaporthe grisea pth11p is a novel plasma membrane protein that mediates appressorium differentiation in response to inductive substrate cues. Plant Cell 11: 2013–2030. 10521529 10.1105/tpc.11.10.2013 PMC144101

[56] Gibson SK, Gilman AG (2006) Giα and Gβ subunits both define selectivity of G protein activation by α2-adrenergic receptors. Proceedings of the National Academy of Sciences of the United States of America 103: 212–217. 16371464 10.1073/pnas.0509763102 PMC1325004

[57] Kutateladze TG (2010) Translation of the phosphoinositide code by PI effectors. Nat Chem Biol 6: 507–513. 20559318 10.1038/nchembio.390 PMC3182472

[58] Gillooly DJ, Morrow IC, Lindsay M, Gould R, Bryant NJ, et al (2000) Localization of phosphatidylinositol 3-phosphate in yeast and mammalian cells. EMBO J 19: 4577–4588. 10970851 10.1093/emboj/19.17.4577 PMC302054

[59] Steinberg G, Schliwa M, Lehmler C, Bolker M, Kahmann R, et al (1998) Kinesin from the plant pathogenic fungus Ustilago maydis is involved in vacuole formation and cytoplasmic migration. J Cell Sci 111 ((Pt 15)) 2235–2246. 9664045 10.1242/jcs.111.15.2235

[60] Wedlich-Soldner R, Bolker M, Kahmann R, Steinberg G (2000) A putative endosomal t-SNARE links exo- and endocytosis in the phytopathogenic fungus Ustilago maydis. EMBO J 19: 1974–1986. 10790364 10.1093/emboj/19.9.1974 PMC305698

[61] Breakspear A, Pasquali M, Broz K, Dong Y, Kistler HC (2011) Npc1 is involved in sterol trafficking in the filamentous fungus Fusarium graminearum. Fungal Genet Biol 48: 725–730. 21397712 10.1016/j.fgb.2011.03.001

[62] Clergeot P-H, Gourgues M, Cots J, Laurans F, Latorse M-P, et al (2001) PLS1, a gene encoding a tetraspanin-like protein, is required for penetration of rice leaf by the fungal pathogen Magnaporthe grisea. Proceedings of the National Academy of Sciences 98: 6963–6968. 10.1073/pnas.111132998 PMC34461 11391010

[63] Shoji JY, Arioka M, Kitamoto K (2006) Vacuolar membrane dynamics in the filamentous fungus Aspergillus oryzae. Eukaryot Cell 5: 411–421. 16467481 10.1128/EC.5.2.411-421.2006 PMC1405889

[64] Slessareva JE, Routt SM, Temple B, Bankaitis VA, Dohlman HG (2006) Activation of the phosphatidylinositol 3-kinase Vps34 by a G protein alpha subunit at the endosome. Cell 126: 191–203. 16839886 10.1016/j.cell.2006.04.045

[65] Takegawa K, DeWald DB, Emr SD (1995) Schizosaccharomyces pombe Vps34p, a phosphatidylinositol-specific PI 3-kinase essential for normal cell growth and vacuole morphology. Journal of Cell Science 108: 3745–3756. 8719881 10.1242/jcs.108.12.3745

[66] Vlahos CJ, Matter WF, Hui KY, Brown RF (1994) A specific inhibitor of phosphatidylinositol 3-kinase, 2-(4-morpholinyl)-8-phenyl-4H-1-benzopyran-4-one (LY294002). Journal of Biological Chemistry 269: 5241–5248. 8106507

[67] Lachmann J, Ungermann C, Engelbrecht-Vandre S (2011) Rab GTPases and tethering in the yeast endocytic pathway. Small Gtpases 2: 182–186. 21776422 10.4161/sgtp.2.3.16701 PMC3136951

[68] Patkar RN, Suresh A, Naqvi NI (2010) MoTea4-mediated polarized growth is essential for proper asexual development and pathogenesis in Magnaporthe oryzae. Eukaryot Cell 9: 1029–1038. 20472691 10.1128/EC.00292-09 PMC2901665

[69] Czymmek KJ, Bourett TM, Shao Y, DeZwaan TM, Sweigard JA, et al (2005) Live-cell imaging of tubulin in the filamentous fungus Magnaporthe grisea treated with anti-microtubule and anti-microfilament agents. Protoplasma 225: 23–32. 15868210 10.1007/s00709-004-0081-3

[70] Horio T, Oakley BR (2005) The role of microtubules in rapid hyphal tip growth of Aspergillus nidulans. Mol Biol Cell 16: 918–926. 15548594 10.1091/mbc.E04-09-0798 PMC545922

[71] Rink J, Ghigo E, Kalaidzidis Y, Zerial M (2005) Rab conversion as a mechanism of progression from early to late endosomes. Cell 122: 735–749. 16143105 10.1016/j.cell.2005.06.043

[72] Gross JC, Chaudhary V, Bartscherer K, Boutros M (2012) Active Wnt proteins are secreted on exosomes. Nat Cell Biol 14: 1036–1045. 22983114 10.1038/ncb2574

[73] Neves SR, Tsokas P, Sarkar A, Grace EA, Rangamani P, et al (2008) Cell Shape and Negative Links in Regulatory Motifs Together Control Spatial Information Flow in Signaling Networks. Cell 133: 666–680. 18485874 10.1016/j.cell.2008.04.025 PMC2728678

[74] Vandamme J, Castermans D, Thevelein JM (2012) Molecular mechanisms of feedback inhibition of protein kinase A on intracellular cAMP accumulation. Cell Signal 24: 1610–1618. 22522182 10.1016/j.cellsig.2012.04.001

[75] Ballon DR, Flanary PL, Gladue DP, Konopka JB, Dohlman HG, et al (2006) DEP-domain-mediated regulation of GPCR signaling responses. Cell 126: 1079–1093. 16990133 10.1016/j.cell.2006.07.030

[76] Ramanujam R, Yishi X, Liu H, Naqvi NI (2012) Structure-Function Analysis of Rgs1 in Magnaporthe oryzae: Role of DEP Domains in Subcellular Targeting. PLoS One 7: e41084. 22927898 10.1371/journal.pone.0041084 PMC3426613

[77] Zhou X, Zhang H, Li G, Shaw B, Xu JR (2012) The Cyclase-Associated Protein Cap1 Is Important for Proper Regulation of Infection-Related Morphogenesis in Magnaporthe oryzae. PLoS Pathog 8: e1002911. 22969430 10.1371/journal.ppat.1002911 PMC3435248

[78] Khang CH, Berruyer R, Giraldo MC, Kankanala P, Park SY, et al (2010) Translocation of Magnaporthe oryzae effectors into rice cells and their subsequent cell-to-cell movement. Plant Cell 22: 1388–1403. 20435900 10.1105/tpc.109.069666 PMC2879738

[79] Janetopoulos C, Jin T, Devreotes P (2001) Receptor-Mediated Activation of Heterotrimeric G-Proteins in Living Cells. Science 291: 2408–2411. 11264536 10.1126/science.1055835

[80] Eaton CJ, Cabrera IE, Servin JA, Wright SJ, Cox MP, et al (2012) The guanine nucleotide exchange factor RIC8 regulates conidial germination through Galpha proteins in Neurospora crassa. PLoS One 7: e48026. 23118921 10.1371/journal.pone.0048026 PMC3485287

[81] Costes SV, Daelemans D, Cho EH, Dobbin Z, Pavlakis G, et al (2004) Automatic and quantitative measurement of protein-protein colocalization in live cells. Biophys J 86: 3993–4003. 15189895 10.1529/biophysj.103.038422 PMC1304300

