# Supplementary material for: The Late Endosomal HOPS Complex Anchors Active G-Protein Signaling Essential for Pathogenesis in Magnaporthe oryzae
Source: PLoS Pathog. 2013 Aug 1;9(8):e1003527. doi: 10.1371/journal.ppat.1003527 (PMC3731250; doi:10.1371/journal.ppat.1003527)
Supplement: Text S1 — Supporting Figures and Tables: Details about (a) Dynamics of Rgs1-mCherry during growth on a non-inductive surface and in the pth11Δ strain (b) Pearson's coefficient analysis and line scan graphs for colocalization between Rgs1 and specific marker proteins (c) BiFC experiment showing in-vivo interaction between Pth11 and Rgs1 at 2 hpi and controls for the hooking stage (4 hpi) (d) Colocalization analysis between Rgs1 and intracellular organelles (e) Effect of PI3 kinase inhibitor LY294002 on the GFP-FYVE strain (f) Orthologs for early or late endosome specific Rab GTPases in M. oryzae (g) Specific colocalization between Rgs1 and the late endosomal moieties (h) Colocalization between MagAG187S-GFP and Rgs1-mCherry and CMAC stained vacuoles (i) Role of F-actin cytoskeleton in Rgs1 dynamics (j) Colony morphology, aerial growth and disease causing ability of the vps39Δ strain (k) Subcellular localization of Rgs1-mC (24 hpi) and during in-planta growth (36 hpi) (l) Characterization and western blot analysis of strains used in this study (m) Characterization, quantification of conidiation and appressorial function of the strains used in this study (n) Oligonucleotide primers used in this study. (PDF) [file ppat.1003527.s001.pdf]

SUPPORTING INFORMATION

Figure S1: Live cell imaging of Rgs1-mCherry on non-inductive surface and in *pth11*Δ strain. (A) Time-course sequence of Rgs1-mC at 4 hpi on a non-inductive surface (1% agar). Rgs1-mCherry (pseudocolored yellow) fails to localize to tubulo-vesicular compartments. Arrowheads indicate small puncta within the germ tube and a prominent vacuolar signal. Time elapsed (seconds) is indicated. Scale bar equals 10 μm. Images are maximum intensity Z-projections of confocal stacks of five slices, 0.5 μm each. **(B)** Subcellular localization of Rgs1-mCherry in the *pth11*Δ strain at 2 and 4 hpi on an inductive surface. Rgs1-mC is predominantly targeted to the vacuole (arrowhead, lower panel at the bottom) in the *pth11*Δ at 4 hpi, unlike in the control. DIC is shown on the left; Rgs1-mC panel, pseudocolored yellow, is on the right. Images represent single plane images captured on a confocal microscope Scale bar, 10 μm.

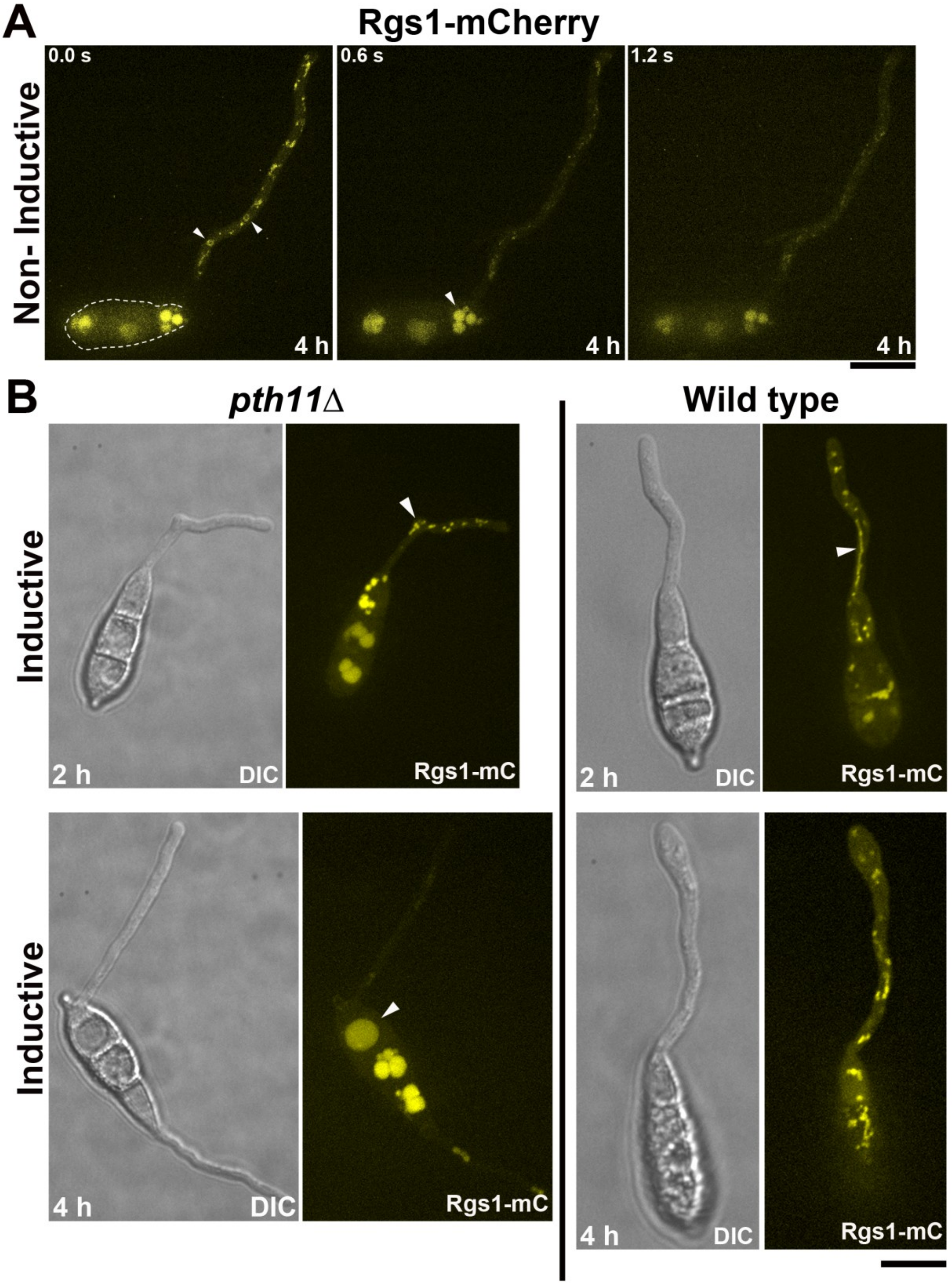
A
Rgs1-mCherry
Non- Inductive
0.0 s
0.6 s
1.2 s
4 h
4 h
4 h
B
pth11Δ
Wild type
Inductive
Inductive
2 h
DIC
Rgs1-mC
2 h
DIC
Rgs1-mC
4 h
DIC
Rgs1-mC
4 h
DIC
Rgs1-mC

Figure S2: Pearson's correlation coefficient analysis and line scans for overlap or colocalization between Rgs1 and Rab5, Rab7, $G\alpha_S$ / MagA^{G187S} and FYVE.

(A) Bar graph illustrating the quantification of the mean Pearson's colocalization coefficient for early endosomal marker Rab5 (n = 5 cells, 47 time points), late endosomal marker Rab7 (n = 6 cells, 80 time points), active MagA^{G187S} (n = 7, 44 time points) or the FYVE-GFP (n = 3 cells, 44 time points) with Rgs1. Error bars correspond to the standard error of the mean.

(**B)** Line scan graphs of Rgs1-mCherry and GFP tagged endosomal markers Rab7, FYVE, and Rab5**.** Representative intensity profiles were obtained for each channel from a single slice along the indicated axes (X and Y) and from the corresponding transverse section of the entire stack (Z) using Fiji software.

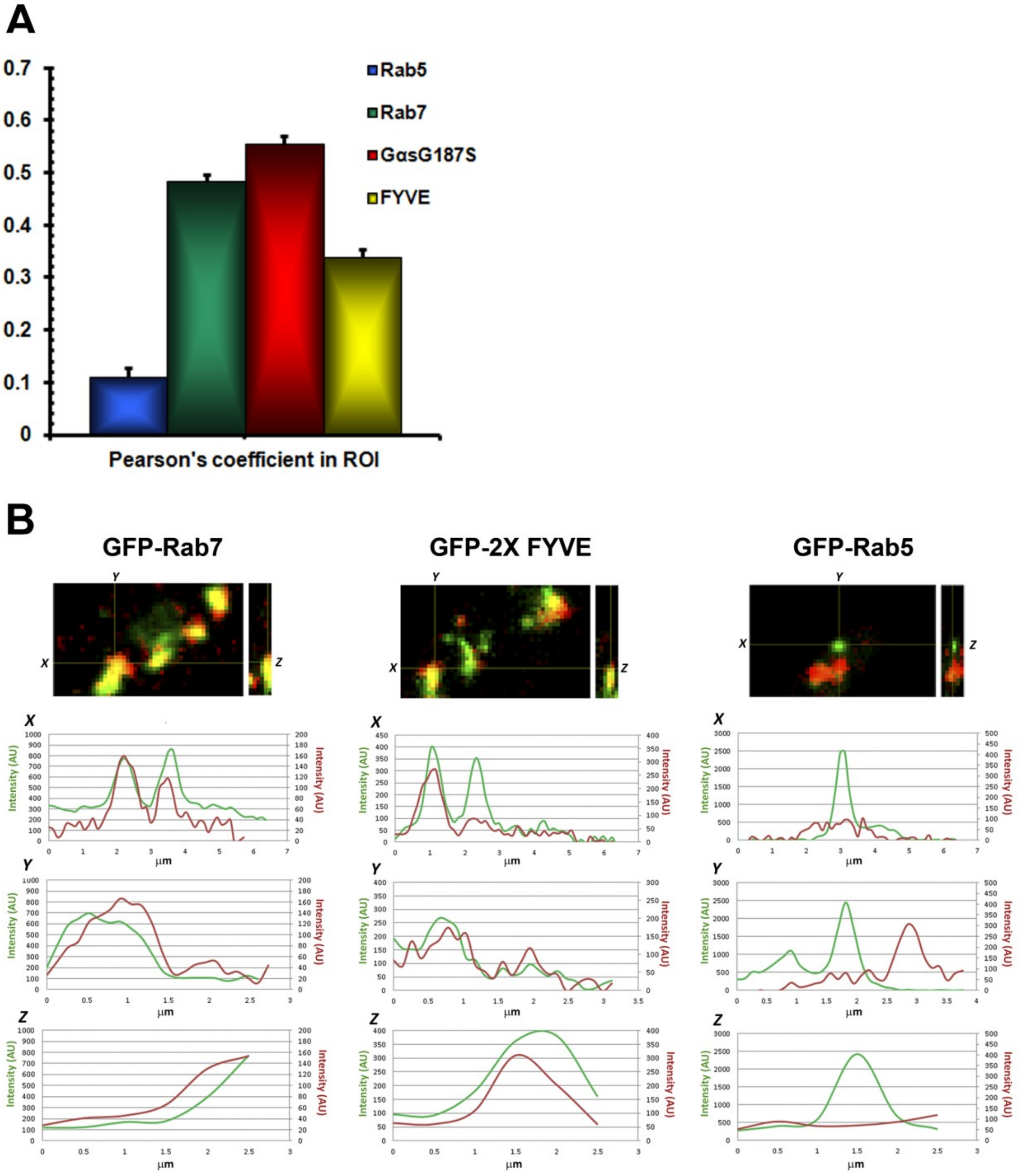
A
0.7
0.6
0.5
0.4
0.3
0.2
0.1
0
Rab5
Rab7
GαsG187S
FYVE
Pearson's coefficient in ROI
B
GFP-Rab7
GFP-2X FYVE
GFP-Rab5
X
Y
Z
Intensity (AU)
μm

Figure S3: BiFC/Split YFP experiment showing the *in vivo* interaction between Pth11 and Rgs1. (A) Images from a BiFC experiment illustrating the interaction between Pth11-nYFP and Rgs1-cYFP at 2hpi on an inductive surface, as ascertained by YFP signal. Asterisk indicates the likely interaction of the two proteins at the plasma membrane. Micrographs represent single plane images captured on a confocal microscope. Scale bar, 10 µm. **(B)** Images showing lack of YFP signal in the negative controls at 4hpi, i.e., in strains expressing either Pth11-nYFP or Rgs1-cYFP alone. Corresponding DIC images shown on the left in each instance. Scale bar, 10 µm.

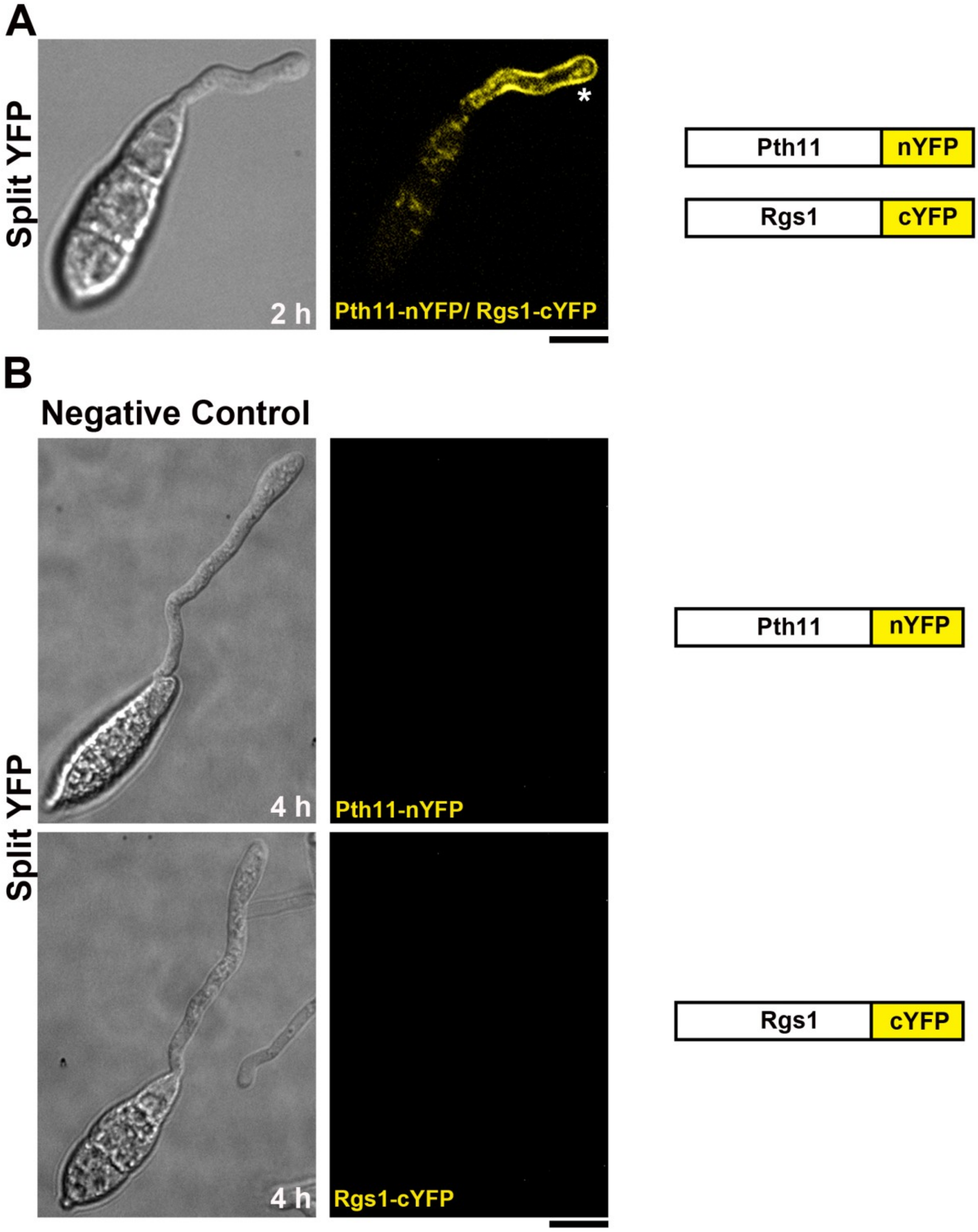
A
Split YFP
2 h
Pth11-nYFP/ Rgs1-cYFP
*
Pth11
nYFP
Rgs1
cYFP
B
Negative Control
Split YFP
4 h
Pth11-nYFP
Pth11
nYFP
4 h
Rgs1-cYFP
Rgs1
cYFP

Figure S4: Colocalization of Rgs1-mCherry with endolysosomal compartments, mitochondria and vacuoles. (A) Panel portraying the partial colocalization between Lysotracker green (LTG) and Rgs1-mCherry on an inductive surface at 4 hpi. Arrowheads and the inset highlight the structures containing LTG and Rgs1-mcherry, Scale bar, 10 μm. **(B)** Rgs1-mC does not colocalize with filamentous mitochondria (mito-GFP). Arrowhead indicates the region magnified on the extreme right highlighting distinct Rgs1 structures and mitochondria. Images are maximum intensity Z-projections of confocal stacks of 5 slices, 0.5 μm each. Scale bar equals 10 μm. **(C)** Rgs1-mC tubulo-vesicular compartments do not colocalize prominently with CMAC-stained vacuoles (arrowhead). The conidial vacuole contains both CMAC and Rgs1. Rgs1-mC, top panel; CMAC, bottom panel. Images are single plane images captured on a confocal microscope. Scale bar 10 μm.

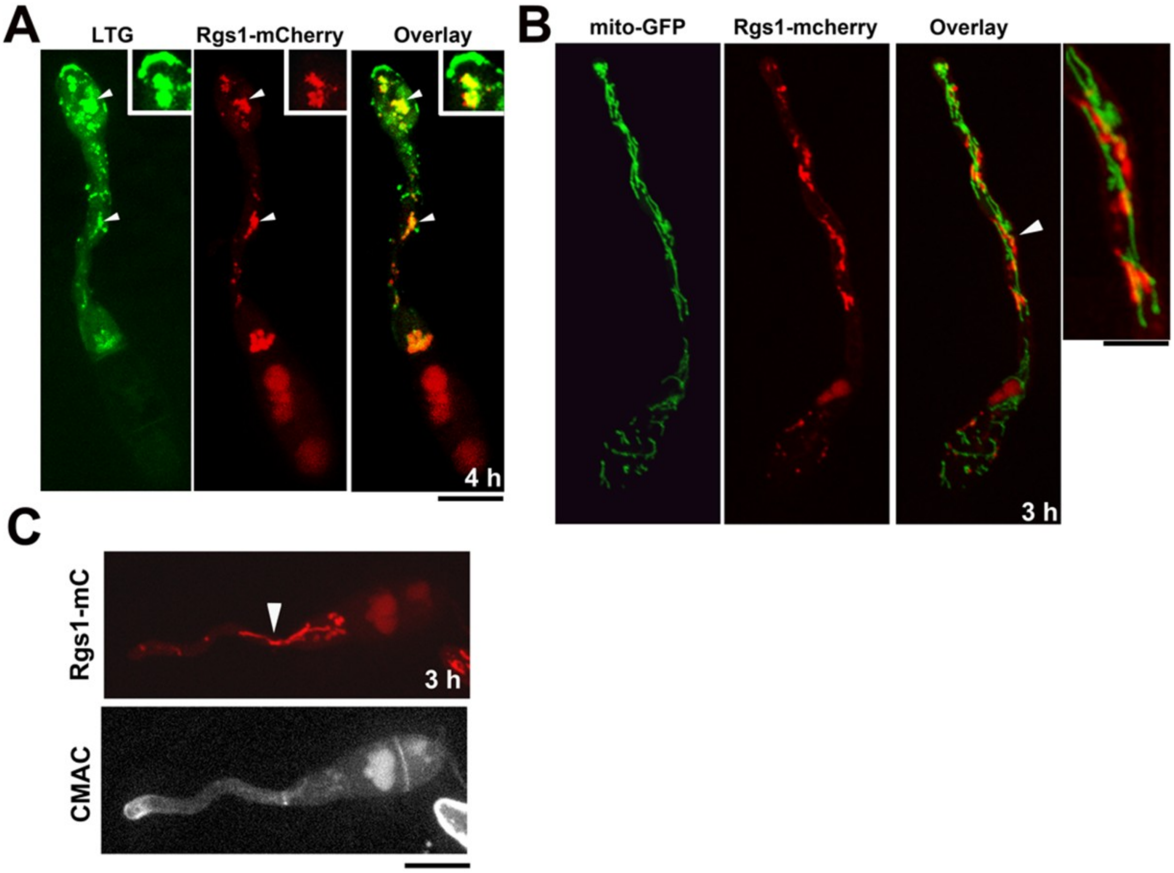

Figure S5: Effect of PI3 kinase inhibitor LY294002 on the *GFP-FYVE* strain. The GFP-2X-FYVE strain was treated with the PI3 kinase inhibitor LY294002, 4 hpi. The treated samples showed enlarged vacuolar compartments and the accumulation of the FYVE probe therein, unlike the DMSO treated control. Images are single plane images captured on a confocal microscope. Scale bar, 10 μm.

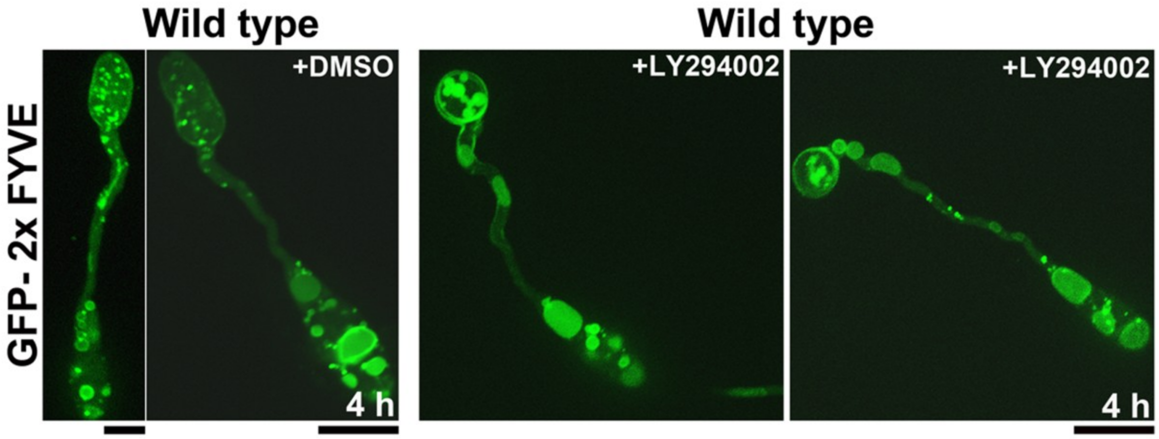

Figure S6: Comparative sequence alignment of Rab5 and Rab7 orthologs in *M. oryzae*. **(A)** Amino acid sequences from Rab5 (Vps21) and Rab7 (Ypt7) orthologs from *M. oryzae*, *N. crassa*, *S. cerevisiae* and *S. pombe*. All the orthologs contain the highly conserved switch regions. **(B)** Confocal images depicting early endosomal (GFP-Rab5) compartments marked by FM4-64. The arrowheads highlight a few such structures amongst a vast majority of endosomes. Images are single plane images captured on a confocal microscope. Scale bar, 10 µm.

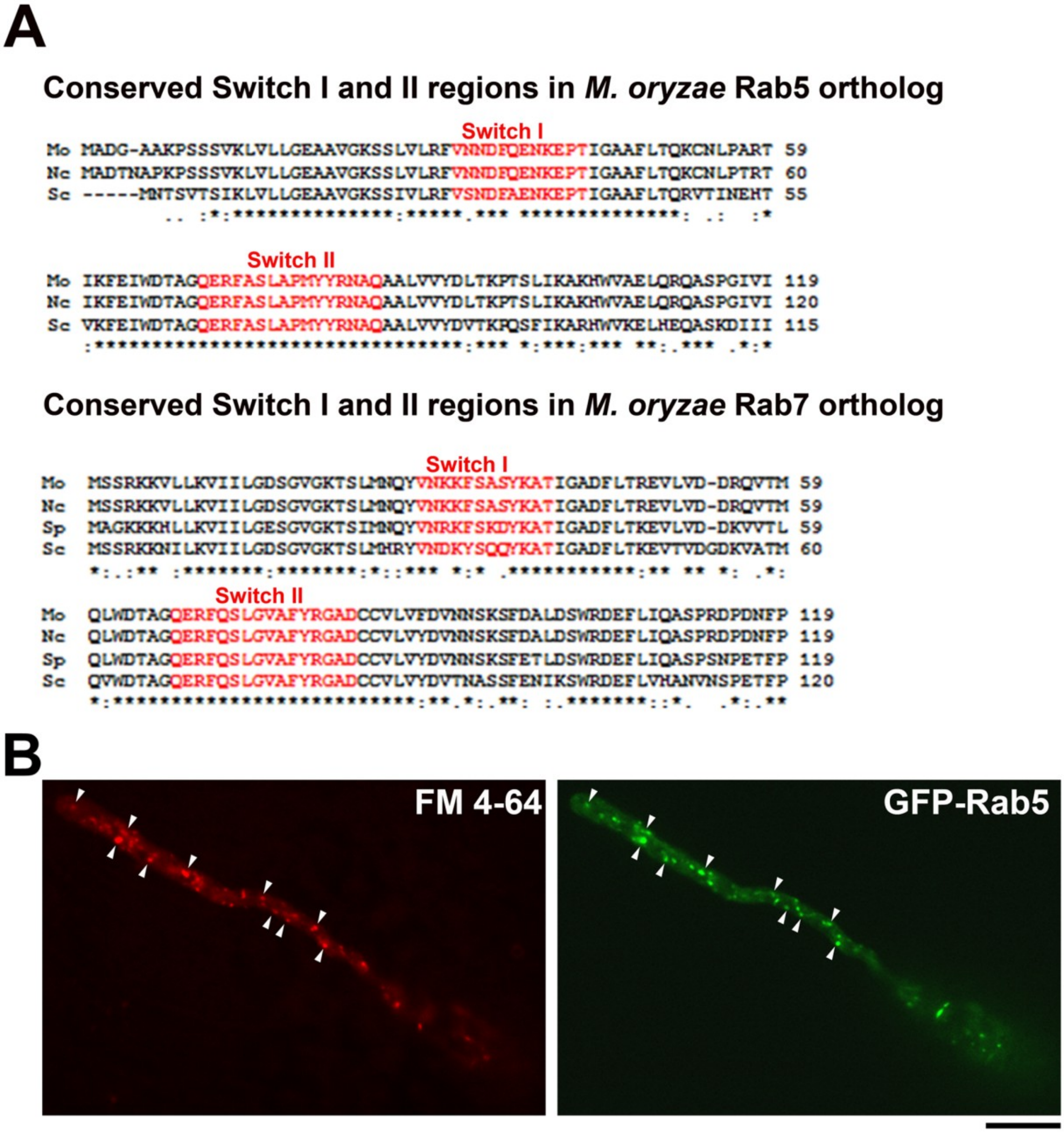

Figure S7: Colocalization of Rgs1-mCherry with Rab7 marked late endosomes. GFP-Rab7 marked late endosomes associate with Rgs1-mCherry structures at the hooking stage. The arrowhead marks the regions magnified in the inset. The yellow asterisk highlights the same compartment in the inset and the corresponding 3D surface rendering. Images are maximum intensity Z-projections of confocal stacks of five slices of 0.5 µm each. Scale bar, 10 µm. See also Video S2.

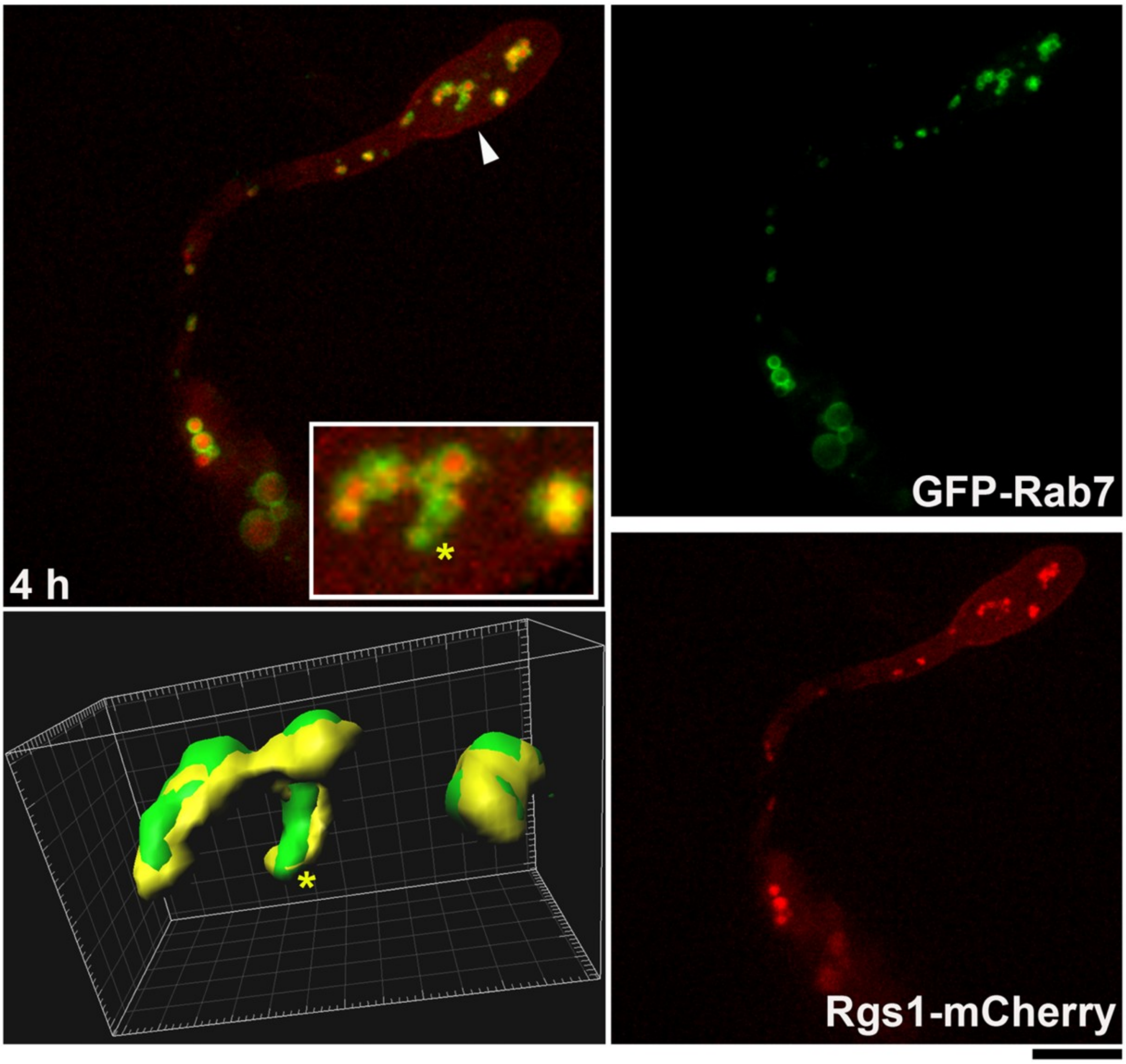

Figure S8: MagA^{G187S}-GFP and Rgs1-mCherry compartments in the germ tube are distinct structures that are not positive for CMAC. (A and **B)** Images from a co-staining experiment between MagA^{G187S}-GFP or Rgs1-mCherry expressing strains with vacuole specific dye CMAC. In both cases, GFP (MagA^{G187S}) and mCherry (Rgs1) positive compartments within the developing appressorium (4 hpi) were distinct and failed to colocalize with CMAC. Co-staining was, although, apparent in vacuoles present in the conidium (arrowhead). Images are single plane images captured on a confocal microscope. Scale bar equals, 10μm.

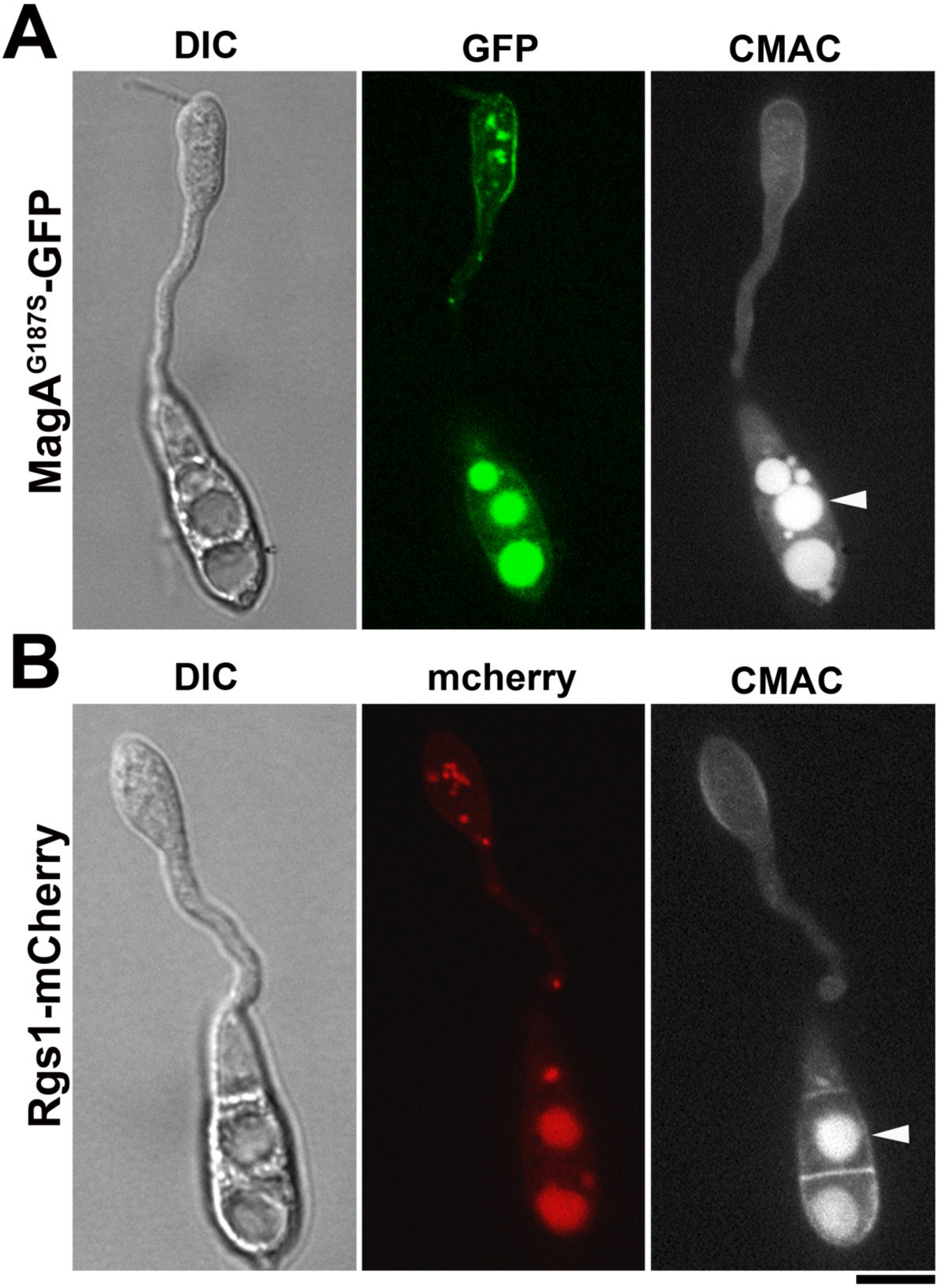
A
DIC
GFP
CMAC
MagA^{G187S}-GFP
B
DIC
mcherry
CMAC
Rgs1-mCherry

Figure S9: Rgs1 compartments utilize microtubules and not the F-actin cytoskeleton for trafficking within the germ tubes. (A) Images depicting Rgs1-mCherry (red) structures coincident with microtubule tracks, marked by GFP-α tubulin. **(B)** Images from a time-lapse movie of Rgs1-mCherry (pseudocolored yellow) strain treated with LatA at 4 hpi. The arrowheads highlight Rgs1-mCherry containing tubulo-vesicular compartments. The time elapsed in seconds is depicted. All images are maximum intensity Z-projections of confocal stacks of five slices (0.5 μm each). Scale bar, 10 μm.

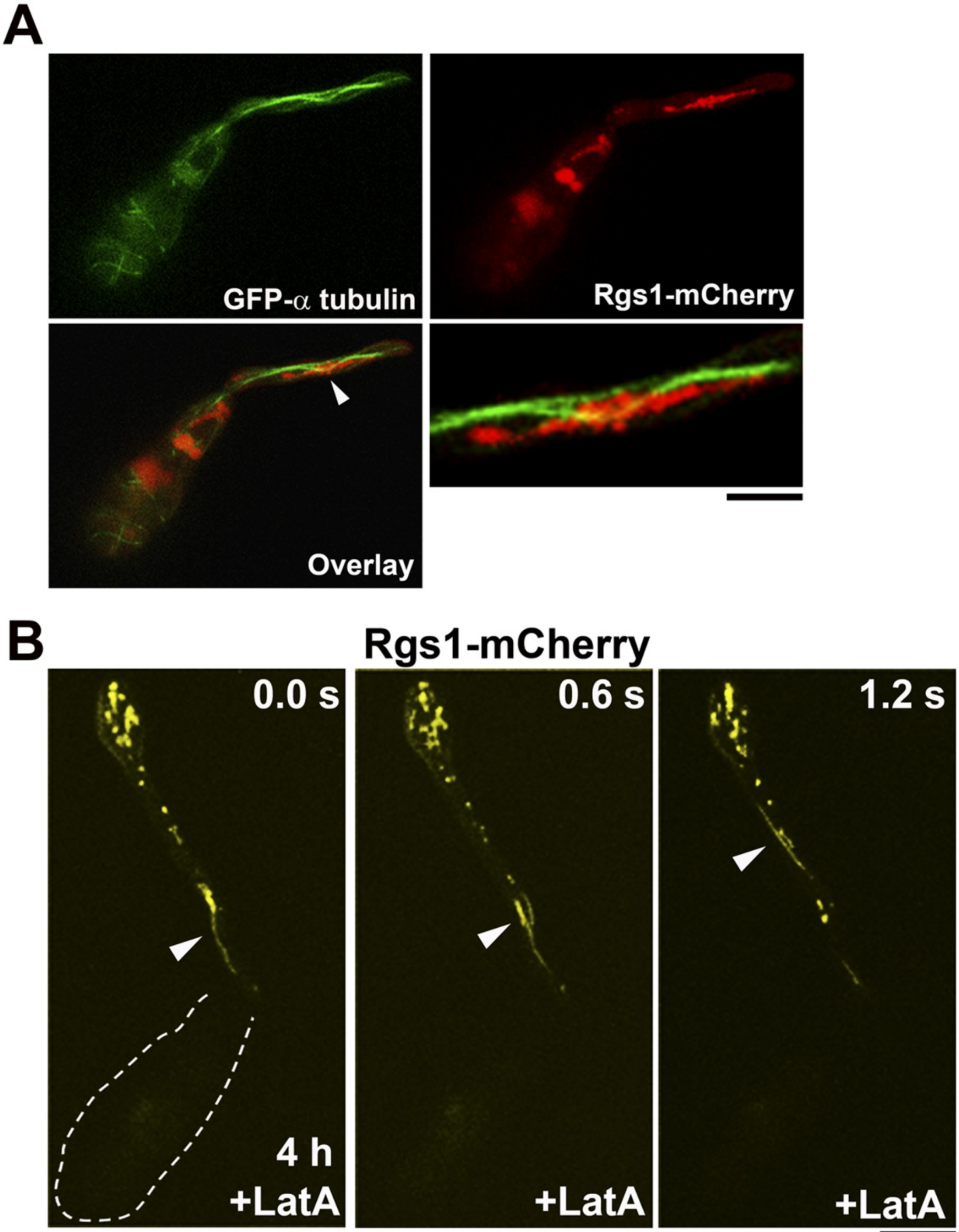
A
GFP-α tubulin
Rgs1-mCherry
Overlay
B
Rgs1-mCherry
0.0 s
0.6 s
1.2 s
4 h
+LatA
+LatA
+LatA

Figure S10: Characterization and infection assay using the *vps39*Δ strain.

(A) Morphology of *vps39*Δ strain. The upper panels are photographs of the indicated strains taken after a week of growth on prune agar. The lower panels depict medial cross-sections of the corresponding colonies, showing aerial hyphal growth. **(B)** Infection assay on barley leaf ex-plants. Barley leaves were inoculated in triplicate with the specified number of conidia per inoculation site. Disease symptoms were evaluated a week post inoculation and compared to the wild type. **(C)** Bar graphs depicting conidiation and appressorial function in the *vps39*Δ. Conidia from the indicated strains were harvested and quantified after growth in the dark on prune agar for 24 h, followed by exposure to constant illumination for 7 days. Data represents mean ± SE of three independent replicates for individual strains. The wild type served as a control. For quantification of host penetration efficiency, the harvested conidia were inoculated on barley leaf explants and the penetration pegs quantified 24 hpi, after staining with aniline blue, that marks host derived callose deposits. The wild type served as a control. Data represents mean ± SE of three independent replicates for individual strains.

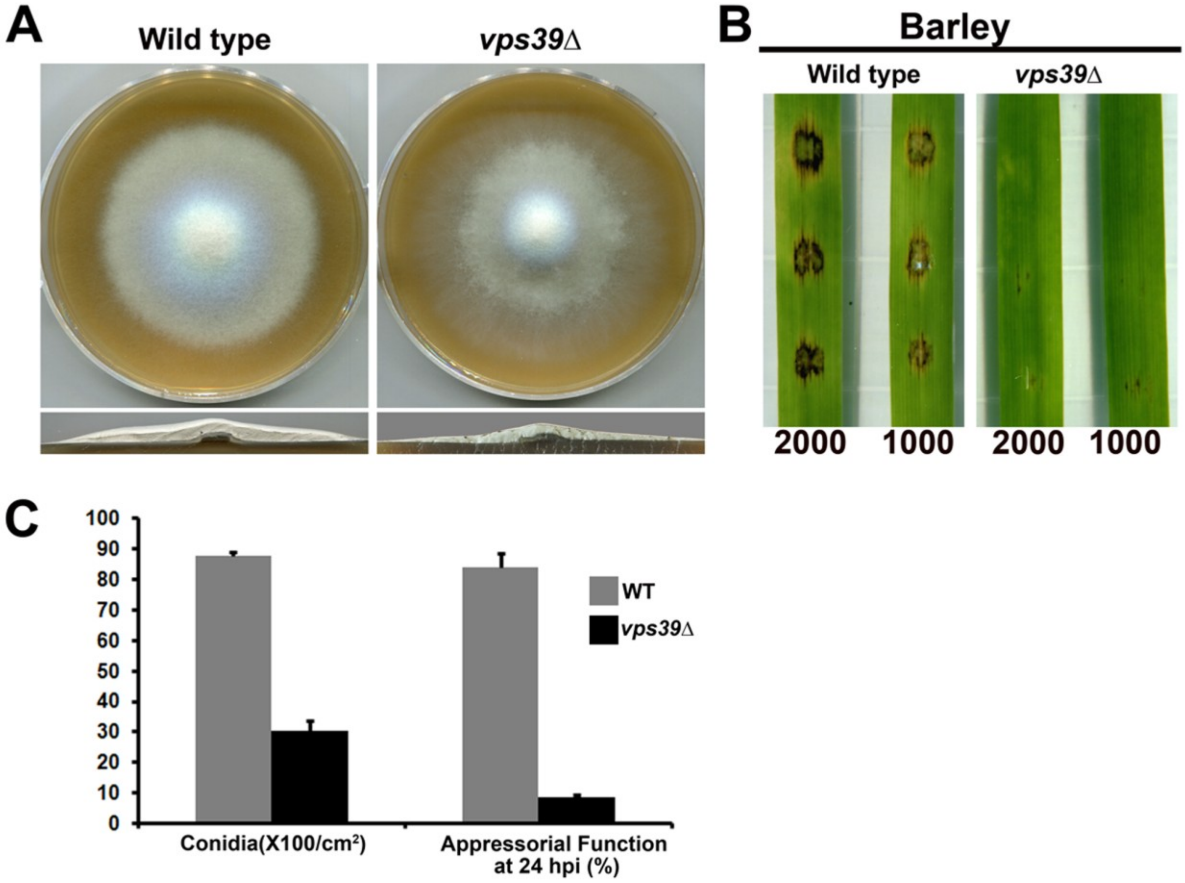
A
Wild type
vps39Δ
B
Barley
Wild type
vps39Δ
2000
1000
2000
1000
C
100
90
80
70
60
50
40
30
20
10
0
WT
vps39Δ
Conidia(X100/cm²)
Appressorial Function at 24 hpi (%)

Figure S11: Rgs1-mC is predominantly vacuolar in mature appressoria and infection hyphae. (A) Rgs1-mCherry expressing strain was inoculated on inductive surface and imaged after 24 hpi. Micrographs are single plane images captured on a confocal microscope. Scale bar, 10 µm. **(B)** Conidia from the Rgs1-mCherry strain were inoculated on rice leaf sheath and imaged 36 hpi. Vacuoles are predominantly found in the infectious hyphae (asterisk).Images are single plane images captured on a confocal microscope. Scale bar, equals 10 µm.

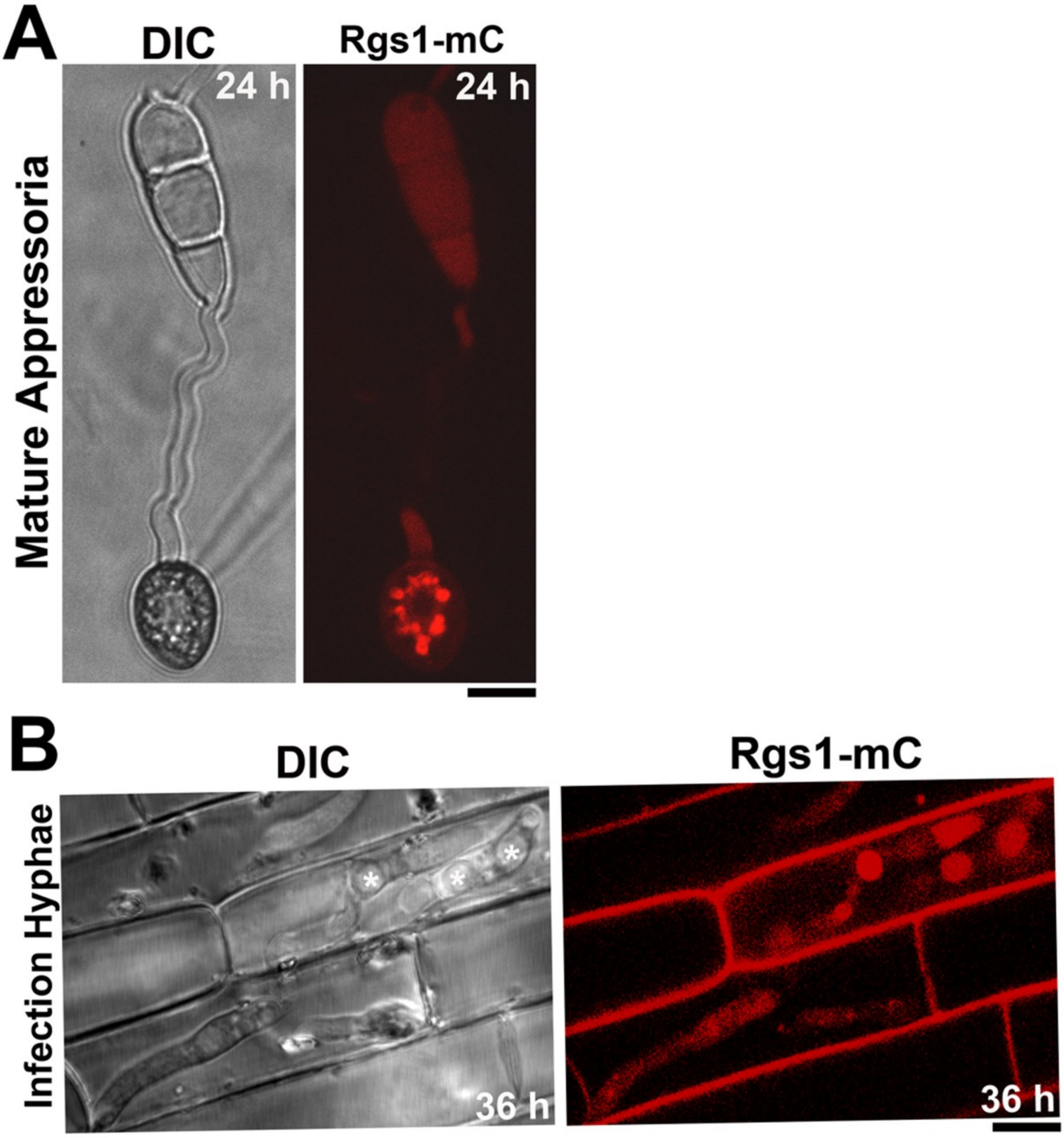

Figure S12: Characterization of strains used in the study to verify biological functionality and integrity of the fusion proteins. All the tagged strains used in this study for imaging purposes were characterized for phenotypic defects and compared to the wild-type strain. **Left:** The upper panels depict the colony morphology after one week of growth on prune agar. The lower panels are medial cross sections of the same colony. **Center:** Conidia were harvested from the relevant strains and inoculated on inductive as well as non-inductive surfaces and imaged after 24 hpi. Images are single plane images captured by DIC on a confocal microscope. Scale bar, 10 µm. **Right:** The conidia were inoculated in triplicate with the specified number of spores per inoculation site and the disease symptoms evaluated after a week. **Far right:** Total protein was extracted from the indicated as well as the relevant control strains and subjected to SDS-PAGE and western blot analysis. The asterisk indicates the specific band of interest.

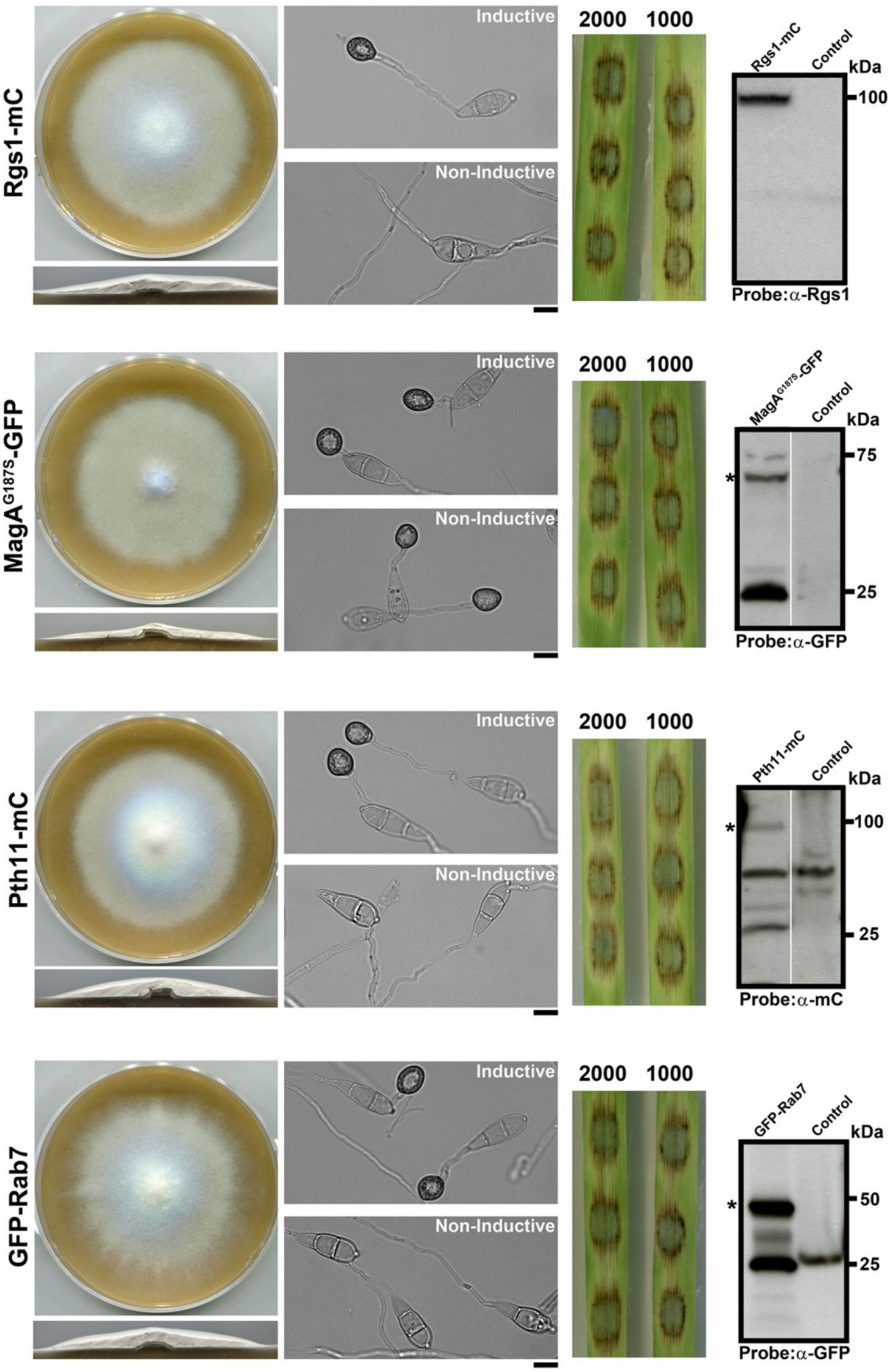

Rgs1-mC
Inductive
Non-Inductive
2000
1000
Rgs1-mC
Control
kDa
100
Probe:α-Rgs1
MagA^G187S-GFP
Inductive
Non-Inductive
2000
1000
MagA^G187S-GFP
Control
kDa
75
25
*
Probe:α-GFP
Pth11-mC
Inductive
Non-Inductive
2000
1000
Pth11-mC
Control
kDa
100
25
*
Probe:α-mC
GFP-Rab7
Inductive
Non-Inductive
2000
1000
GFP-Rab7
Control
kDa
50
25
*
Probe:α-GFP

Figure S13: Characterization, comparative analysis of conidiation and appressorial function of the strains used in this study. (A) The panel on the top shows the morphology of the colony after one week of growth on prune agar. The lower panels are medial cross sections of the colony showing aerial growth. Harvested conidia were inoculated on inductive and non-inductive surfaces and images captured 24 hpi. The conidia were used to carry out infection assays and the disease symptoms evaluated 7 days after inoculation. Protein lysates from the tagged as well as appropriate control strains were resolved on SDS-PAGE and analyzed by western blotting. The asterisks specify the band of interest. **(B)** Conidia were harvested and quantified after the tagged strains were grown in the dark on prune agar for 24 h, followed by exposure to constant illumination for 7 days. Data represents mean ± SE of three independent replicates for individual strains. The wild type served as a control. **(C)** Quantification of host penetration efficiency. Conidia from the indicated strains were inoculated on barley leaf explants and the penetration pegs quantified 24 hpi, after staining with aniline blue (that mark specific host callose deposits). The wild type served as a control. Data represents mean ± SE of three independent replicates for individual strains.

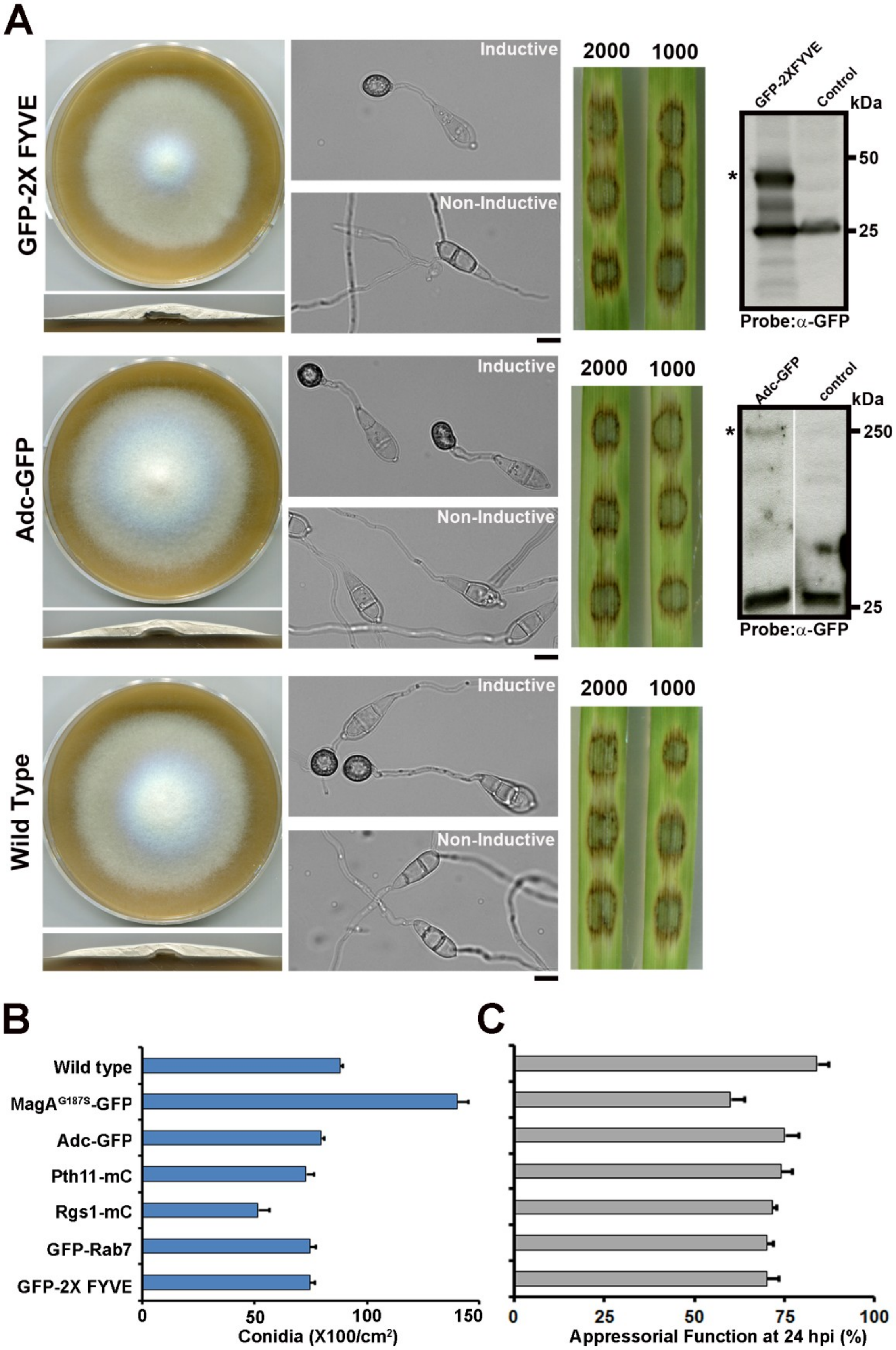
A
GFP-2X FYVE
Inductive
Non-Inductive
2000
1000
GFP-2XFYVE
Control
kDa
50
25
*
Probe:α-GFP
Adc-GFP
Inductive
Non-Inductive
2000
1000
Adc-GFP
control
kDa
250
25
*
Probe:α-GFP
Wild Type
Inductive
Non-Inductive
2000
1000
B
Wild type
MagA G187S-GFP
Adc-GFP
Pth11-mC
Rgs1-mC
GFP-Rab7
GFP-2X FYVE
0
50
100
150
Conidia (X100/cm2)
C
0
25
50
75
100
Appressorial Function at 24 hpi (%)

Table S1: Sequences of oligonucleotide primers used for PCR amplification and molecular cloning. Restriction endonuclease sites introduced for cloning are highlighted in lower case.

Gene amplified	Oligonucleotide Sequence of primers (5' – 3')	Enzyme site
RGS1	CAGAGAgaattcATGGACGACACCTCCCGCC (F)	*EcoR*I
	CAGAGAcccgggTAACCGTTGCGAGCGGCTT (R)	*Sma*I
mCherry	CAGAGAcccgggATGGTGAGCAAGGGCGAGG (F)	*Sma*I
	CAGAGAggatccTTACTTGTACAGCTCGTCCATGCCG (R)	*BamH*I
eGFP	CAGAGAccatggTGAGCAAGGGCGAGGAGCTGT (F)	*Nco*I
	CAGAGAcccgggCTTGTACAGCTCGTCCAT (R)	*Sma*I
TrpC terminator	CAGAGAggatcc/cccgggACTTAACGTTACTGAAATCATCA (F)	*BamH*I/*Sma*I
	CAGAGAtctaga/ggatccCGAGCCCTCTAAACAAGTGT (R)	*Xba*I/ *BamH*I
Rab5/ Vps21	CAGAGAcccgggTCCGGAGGAGGAGGATCCGCCGACGG AGCCGCCAAAC (F)	*Sma*I
	CAGAGAggattcTCAACACGCGCATCCATCC (R)	*BamH*I
Rab7/ Ypt7	CAGAGAcccgggTCCGGAGGCGGTGGCTCGTCCAGAAA GAAGGTTCT (F)	*Sma*I
	CAGAGAcccgggTTAGCAGGCGCATCCATC (R)	*Sma*I

α- Tub	CAGAGAcccgggTCCGGAGGAGGAGGATCCCGCGAGATT	*Sma*I
	ATCAGCATC (F)	*Sma*I
	CAGAGAcccgggCTAGTACTCCGCCTCAATAC (R)	
		*EcoR*I
MagA	GAGCAGgaattcTCCCCACTCCGACGACGT (F)	
UP	TCACTCacgcgtAGACCCTCCGCCACCACTCGACTCG	*Mlu*I
	ACCATGTAGTC (R)	
		*Hpa*I
MagA	GAGCAGgttaacAGCGGCGGAGGTGGCTCGGGAC	
DS	CACAGGCAGAGATAG (F)	*Hind*III
	GAGCTGaagcttAACATCGTTGCCTCCAGAG (R)	
Vps39	CAGAGAgaattcGAGGATAAGCCTTGTGAAG (F)	*EcoR*I
UP	CAGAGAggatccTGCGACGTCGGGCTCGCG (R)	*BamH*I
Vps39	CAGAGActgcagGCTCGGTGTTGAGAGTTGAC (F)	*Pst*I
DS	CAGAGAaagcttCTGTTACCCGTTCAGCCGTC (R)	*Hind*III
